# Restriction estimates of $\varepsilon$-removal type for *k*-th powers and paraboloids

**DOI:** 10.1007/s00208-018-1650-7

**Published:** 2018-02-23

**Authors:** Kevin Henriot, Kevin Hughes

**Affiliations:** 1 Department of Mathematics, University of British Columbia, Room 121, 1984 Mathematics Road, Vancouver, BC V6T 1Z2 Canada; 2 School of Mathematics, University of Bristol, Howard House, Queens Avenue, Bristol, BS8 1SN UK; 3 The Heilbronn Institute for Mathematical Research, Bristol, UK

**Keywords:** 11P05, 11P55, 42B25, 42B37

## Abstract

We obtain restriction estimates of $\varepsilon$-removal type for the set of *k*-th powers of integers, and for discrete *d*-dimensional surfaces of the form

$$\begin{aligned} \{ (n_1,\dots ,n_d,n_1^k + \cdots + n_d^k) \,:\, |n_1|,\dots ,|n_d| \leqslant N \}, \end{aligned}$$

which we term ‘*k*-paraboloids’. For these surfaces, we obtain a satisfying range of exponents for large values of *d*, *k*. We also obtain estimates of $\varepsilon$-removal type in the full supercritical range for *k*-th powers and for *k*-paraboloids of dimension $d < k(k-2)$. We rely on a variety of techniques in discrete harmonic analysis originating in Bourgain’s works on the restriction theory of the squares and the discrete parabola.

## Introduction

We are interested in restriction theorems for discrete surfaces in $\mathbb {Z}^d$. We restrict our attention to parametric surfaces of the form

1.1 $$\begin{aligned} S = \{ \mathbf {P}(\mathbf {n}) \,:\, \mathbf {n}\in [-N,N]^d \}, \end{aligned}$$

where $\mathbf {P}= (P_1,\dots ,P_r)$ is a system of *r* integer polynomials in *d* variables, and we assume that the map $\mathbf {P}: \mathbb {Z}^d \rightarrow \mathbb {Z}^r$ is injective for simplicity. When the polynomials $P_1,\dots ,P_r$ have degree $k_1,\dots ,k_r$, we define the total degree of the system $\mathbf {P}$ as $K = k_1 + \cdots + k_r$. We denote the action of the extension operator on a sequence $a : \mathbb {Z}^d \rightarrow \mathbb {C}$ supported on $[-N,N]^d$ by

$$\begin{aligned} F_a^{(\mathbf {P})}(\varvec{\alpha })&= \sum _{\mathbf {n}\in \mathbb {Z}^d} a(\mathbf {n}) e\big ( \mathbf {P}(\mathbf {n}) \cdot \varvec{\alpha }\big )&(\varvec{\alpha }\in \mathbb {T}^r). \end{aligned}$$

The natural restriction conjecture, based on heuristics from the circle method, is that the $\varepsilon$-free estimate

1.2 $$\begin{aligned} \Vert F_a^{(\mathbf {P})} \Vert _p^p \lesssim N^{\frac{dp}{2} - K} \Vert a \Vert _2^p \end{aligned}$$

holds in the supercritical range $p > \tfrac{2K}{d}$, the $\varepsilon$-full estimate

1.3 $$\begin{aligned} \Vert F_a^{(\mathbf {P})} \Vert _q^q \lesssim _\varepsilon N^{\frac{dq}{2} - K + \varepsilon } \Vert a \Vert _2^q \end{aligned}$$

holds at the critical exponent $q = \frac{2K}{d}$, and the subcritical estimate

1.4 $$\begin{aligned} \Vert F_a^{(\mathbf {P})} \Vert _p^p \lesssim _\varepsilon N^{\varepsilon } \Vert a \Vert _2^p \end{aligned}$$

holds for $2 \leqslant p < \frac{2K}{d}$. This conjecture has to be corrected when the discrete surface $\{ \mathbf {P}(\mathbf {x}),\,\mathbf {x}\in \mathbb {Z}^d \}$ contains large special subvarieties, but this does not appear to be the case for the surfaces we study.

In the supercritical range, Bourgain resolved the natural restriction conjecture in the case $\mathbf {P}= (x^2)$ of the squares [2] and in the case $\mathbf {P}= (x,x^2)$ of the 2*D* parabola [3], via discrete versions of the Tomas–Stein argument [25, Chapter 7] and the Hardy–Littlewood circle method. Bourgain and Demeter [4] later established the $\varepsilon$-full estimate (1.3) for arbitrary definite irrational paraboloids $\mathbf {P}= (x_1, \dots , x_d, \theta _1 x_1^2 + \cdots + \theta _d x_d^2)$ with $\theta _i \in (0,1]$ in the full supercritical range $p \geqslant \frac{2(d+2)}{d}$, by developing powerful methods of multilinear harmonic analysis (the indefinite case was later resolved in [5]). In the rational case $\theta _1 = \dots = \theta _d = 1$, the $\varepsilon$-loss can be eliminated via Bourgain’s earlier work [3]. In an important recent work, Killip and Vişan [16] removed the $\varepsilon$-loss for all definite parabolas, using new techniques partly inspired by Bourgain’s [3]. This note relies only on the earlier number-theoretic approach of Bourgain [2, 3], albeit with significant modifications, since it is more adapted to our objective. Indeed, we primarily seek to obtain weaker estimates of the form

1.5 $$\begin{aligned} \int _{|F_a^{(\mathbf {P})}| \geqslant N^{d/2 - \zeta } \Vert a\Vert _2} |F_a^{(\mathbf {P})}|^q \mathrm {d}m\lesssim N^{\frac{dq}{2} - K} \Vert a \Vert _2^q, \end{aligned}$$

for a certain $\zeta > 0$, in the complete supercritical range of exponents $q > \frac{2K}{d}$, or a good approximation thereof. We succeed in doing so for several classes of surfaces generalizing that of the squares and the parabola. Moreover, our precise values of $\zeta$ are obtained by making use of the recent resolution of the Vinogradov mean value conjectures in [26] and [6].

Before introducing these results, we discuss our motivation to seek $\varepsilon$-removal estimates of the form (1.5). Justifying their terminology, these estimates can be used to remove the extraneous factor $N^\varepsilon$ in (1.3), as recalled in Lemma 3.1 below. Methods of multilinear harmonic analysis [4], or even moment bounds exploiting arithmetic information typically produce a factor of this form. While the $N^\varepsilon$ factor is sometimes inconsequential, the sharp estimate (1.2) is often necessary in applications to additive combinatorics. More specifically, restriction estimates are of key importance in the study of linear equations of the form $\sum _{i=1}^s \lambda _i \mathbf {P}(\mathbf {n}_i) = 0$, where the $\lambda _i$ are non-zero integer coefficients summing to zero and the variables $\mathbf {n}_i$ lie in a sparse subset of $\{ 1,\dots , N \}^d$ (or in a sparse subset of $( \mathcal {P}\cap \{ 1,\dots , N \} )^d$, where $\mathcal {P}$ are the prime numbers). When the system of polynomials $\mathbf {P}$ is translation-invariant^1^, this system of equations can be studied via density-increment-based strategies [10, 11, 15, 20] exploiting $L^\infty \rightarrow L^p$ or $L^2 \rightarrow L^p$ restriction estimates for the surface (1.1); we refer to [11] for a more complete discussion. In the general case, one can also analyze such systems by transference-based strategies [7–9] which rely only on $L^\infty \rightarrow L^p$ estimates, although these take a more complicated shape due to the presence of the *W*-trick.

Note also that truncated estimates of the form (1.5) can be completed into full estimates of the form (1.2) for a large enough range of exponents, whenever a subcritical estimate of the form (1.4) is known (which is always the case for $p = 2$). This familiar procedure is recalled in Lemma 3.6 below, but it generally gives a poor range of exponents due to the smallness of the parameter $\zeta$, which is related to Weyl exponents.

The first surface we study is

1.6 $$\begin{aligned} S = \{\, n^k \,:\, n \in \{1,\dots ,N\} \,\}, \end{aligned}$$

corresponding to the system of polynomials $\mathbf {P}= (x^k)$ of total degree *k*, when $k \geqslant 3$ is an integer. In this case we obtain the complete supercritical range of exponents for (1.5) and an incomplete range of exponents for truncated restriction estimates of the form (1.2).

### Theorem 1.1

Let $k \geqslant 3$ and $\tau = \max (2^{1-k},\frac{1}{k(k-1)})$, and write $\mathbf {P}= (x^k)$. The estimate (1.5) holds for any $p > 2k$ and $\zeta < \tau /2$, and the estimate (1.2) holds for $p > 2 + 2 (k - 1) / \tau$.

The proof of this result consists in an adaptation of Bourgain’s argument for squares [2]. We comment in Sect. 5 on the results that can be obtained for arbitrary monomial curves by this approach. It turns out that one only obtains the whole supercritical range for the curve $(n^k)$, due to the lack of efficient majorants of Weyl sums on major arcs in other cases.

Let $R_{s,k}(n)$ denote the number of representations of *n* as a sum of *s* *k*-th powers of integers. Hypothesis *K* of Hardy and Littlewood [24, Section 17] states that $R_{k,k}(n) \lesssim _\varepsilon n^{\varepsilon }$ for $k \geqslant 2$. It is known (and easy to show) for $k = 2$, and while it has been disproved for $k=3$ by Mahler [17], it remains open for $k \geqslant 4$. Under this strong hypothesis, which is far out of reach of current methods, our epsilon-removal estimate implies the full supercritical range of conjectured restriction estimates for *k*-th powers.

### Corollary 1.2

Let $k \geqslant 3$ and write $\mathbf {P}= (x^k)$. If Hypothesis K is true, the estimate (1.2) holds for $p > 2k$.

Fix a dimension $d \geqslant 1$ and a degree $k \geqslant 3$. The next surface we study is the truncated *d*-dimensional *k*-paraboloid embedded in $\mathbb {Z}^{d+1}$

1.7 $$\begin{aligned} S = \{\, (n_1,\dots ,n_d,n_1^k + \cdots + n_d^k) \,,\, n_i \in [-N,N] \cap \mathbb {Z}\,\}, \end{aligned}$$

which is the usual paraboloid when $k = 2$. Our first theorem simplifies the approach of Bourgain for the parabola [3]; the cost of our simplification is that we do not acquire the full supercritical range of $p>\frac{2(d+k)}{d}$, and in particular, we “lose *k* variables” from the critical exponent $\frac{2(d+k)}{d}$.

### Theorem 1.3

Suppose that $d \geqslant 1$ and $k \geqslant 3$, and let $\tau = \max (2^{1-k},\frac{1}{k(k-1)})$. Write also $\mathbf {P}= (x_1,\dots ,x_d,x_1^k + \cdots + x_d^k)$. The truncated estimate (1.5) holds for $\zeta < \frac{d\tau }{2}$ and $p > \frac{2(d+k) + 2k}{d}$, and the estimate (1.2) holds for $p > 2 + \frac{2k}{d\tau }$.

Note that the exponent $\frac{2(d+k)}{d} + \frac{2k}{d}$ approximates the critical exponent $\frac{2(d+k)}{d}$ when the dimension $d+1$ of the ambient space is large with respect to the degree *k* of the paraboloid. In our proof, this reflects the fact that the splitting behavior (7.4) of exponential sums dominates for large dimensions. By adapting the difficult argument of Bourgain [3] in a more direct fashion, we can recover the complete supercritical range of exponents, but only for sufficiently small dimensions, of size roughly less than the square of the degree.

### Theorem 1.4

Suppose that $d \geqslant 1$ and $k \geqslant 3$, and let $\tau = \max \left( 2^{1-k},\frac{1}{k(k-1)}\right)$. Write also $\mathbf {P}= (x_1,\dots ,x_d,x_1^k + \cdots + x_d^k)$, and assume that $d < \frac{k^2 - 2k}{1 - k\tau }$. Then the estimate (1.5) holds for any $\zeta < \frac{d\tau }{2}$ and $p > \frac{2(d+k)}{d}$.

The proof of Theorems 1.3 and 1.4 exploits available bounds on one-dimensional Weyl sums of degree *k*, such as estimates of Weyl type and the Poisson formula on major arcs. The poor quality of known minor arc bounds is the main reason for our relative condition on *d* and *k* in Theorem 1.4. It is a curious feature that in dimension $d = 1$ (say), the approach of Bourgain [3] apparently yields the whole supercritical range for the “sparse” curve $(x,x^k)$. Note that this removes the $\varepsilon$-loss in the restriction estimates of Hu and Li [12, 13] for these curves. We remark also that for very large dimensions, the method of proof of Theorem 1.4 also yields restriction exponents, but the range so obtained is much narrower than that of Theorem 1.3.

We make a last remark about the exponent $\tau$ in Theorems 1.3 and 1.4, which affects dramatically the quality of full restriction estimates we can obtain as corollaries. In those results, one can in fact take $\tau$ to be the largest exponent such that, for all $\alpha \in \mathbb {T}$ such that there exists $q,a \in \mathbb {Z}$ with $N \leqslant q \leqslant N^k$ and $\Vert \alpha - \frac{a}{q} \Vert \leqslant \frac{1}{qN^{k-1}}$,

1.8 $$\begin{aligned} \bigg | \sum _{n=1}^N e(\alpha n^k + n\theta ) \bigg | \lesssim _\varepsilon N^{1 + \varepsilon } \Big ( \frac{1}{q} + \frac{1}{N} + \frac{q}{N^k} \Big )^\tau \end{aligned}$$

uniformly in $\theta \in \mathbb {T}$. For a fixed degree *k*, the best one can hope for $\tau$ is to be 1 / *k*; see e.g. [18, Problem 8, p. 196]. If (1.8) were to hold for all $\tau < 1/k$, then Theorem 1.4 would improve to the full supercritical range in all dimensions. Instead the range in Theorem 1.4 relies on the best known unconditional exponent $\tau$, which is $\tau = \frac{1}{k(k-1)}$ for large values of *k* by Bourgain–Demeter–Guth’s recent resolution of Vinogradov’s mean value conjecture [6], or $\tau = 2^{1-k}$ for small values of *k* by the much simpler Weyl inequality [23] (see Appendix A for more information). Improved bounds on Weyl sums are known for intermediate values of *k*, but they typically take a different shape than (1.8), and therefore we do not try to incorporate them in our argument. In conclusion, it seems that one current limitation of number-theoretic approaches to restriction estimates for surfaces of high degree is the poor quality of known minor arc bounds for Weyl sums. In fact, even optimal Weyl exponents would not allow us to obtain efficient full restriction estimates. Fortunately, results of $\varepsilon$-removal type ignore minor arcs to some extent, hence the efficient ranges in those cases.

## Notation

For functions $f : \mathbb {T}^d \rightarrow \mathbb {C}$ and $g : \mathbb {Z}^d \rightarrow \mathbb {C}$ we define the Fourier transforms of *f* and *g* by $\widehat{f}( \mathbf {k}) = \int _{\mathbb {T}^d} f( \varvec{\alpha }) e( - \varvec{\alpha }\cdot \mathbf {k}) \mathrm {d}\varvec{\alpha }$ and $\widehat{g}( \varvec{\alpha }) = \sum _{\mathbf {n}\in \mathbb {Z}^d} g(\mathbf {n}) e( \varvec{\alpha }\cdot \mathbf {n})$. For a function $h : \mathbb {R}^d \rightarrow \mathbb {C}$ we define the Fourier transform by $\widehat{h}(\varvec{\xi }) = \int _{\mathbb {R}^d} f(\mathbf {x}) e( - \varvec{\xi }\cdot \mathbf {x}) \mathrm {d}\mathbf {x}$. For any function *f* defined on an abelian group, we let $\widetilde{f}(x) = f(-x)$. Given a function $f : \mathbb {R}^d \rightarrow \mathbb {R}$ and two subsets *A*, *B* of $\mathbb {R}^d$, we write $A \prec f \prec B$ when $0 \leqslant f \leqslant 1$ everywhere, $f = 1$ on *A* and $f = 0$ outside *B*. We denote the disjoint union of two sets *A* and *B* by $A \bigsqcup B$.

When $\mathcal {P}$ is a certain property, we let $1_\mathcal {P}$ denote the boolean equal to 1 when $\mathcal {P}$ holds and 0 otherwise, and when *E* is a set we define the indicator function of *E* by $1_E(x) = 1_{x \in E}$. When $p \in [1,+\infty ]$ is an exponent, we systematically denote by $p' \in [1,+\infty ]$ its dual exponent satisfying $\frac{1}{p} + \frac{1}{p'} = 1$. We let $\mathrm {d}m$ denote the Lebesgue measure on $\mathbb {R}^d$, or on $\mathbb {T}^d$ identified with any fundamental domain of the form $[\theta ,1 + \theta )^d$, and we let $\mathrm {d}\Sigma$ denote the counting measure on a discrete set such as $\mathbb {Z}^d$. When $p \in [1,+\infty ]$ is an exponent and $f : \mathbb {T}^d \rightarrow \mathbb {C}$, $\Vert f\Vert _p$ denotes the Lebesgue $L^p(\mathbb {T}^d, \mathrm {d}m)$-norm. Similarly, for $g : \mathbb {Z}^d \rightarrow \mathbb {C}$, $\Vert g\Vert _p$ denotes the $\ell ^p(\mathbb {Z}^d, \mathrm {d}\Sigma )$-norm. However, when $\alpha$ is an element of $\mathbb {T}$ or $\mathbb {R}$, then $\Vert \alpha \Vert$ denotes the distance from $\alpha$ to the nearest integer. For $\alpha \in \mathbb {T},$ we let $\tau _{\alpha }$ denote the translation operator which maps a function $f:\mathbb {T}\rightarrow \mathbb {C}$ to $\tau _{\alpha }f(\theta ):=f(\theta +\alpha )$.

For $q \geqslant 2$ we occasionally use $\mathbb {Z}_q$ as a shorthand for the group $\mathbb {Z}/q\mathbb {Z}$. When *N* is an integer we write $[N] = \{ 1 , \dots , N \}$. Throughout the article, we use the letter $\varepsilon$ generically to denote a constant which can be taken arbitrarily small, and whose value may change in each occurrence.

## Analytic preliminaries

In this section we discuss several standard tools in discrete restriction theory, such as even moment bounds, the epsilon-removal process, and Bourgain’s [2, 3] discrete version of the Tomas–Stein argument [25, Chapter 7] from Euclidean harmonic analysis.

We will often use a smooth weight function $\omega : \mathbb {R}\rightarrow [0,1]$ of the form

3.1 $$\begin{aligned} \omega = \eta \Big ( \frac{\,\cdot \,}{N} \Big ), \qquad \eta \text { Schwarz function such that}\ [-1,1] \prec \eta \prec [-2,2]. \end{aligned}$$

Given a dimension $d \geqslant 1$, we also define the tensorized version

3.2 $$\begin{aligned} \omega _{d}(x_1,\dots ,x_d) :=\omega (x_1) \cdots \omega (x_d). \end{aligned}$$

Consider now an injective map $\mathbf {P}: \mathbb {Z}^d \rightarrow \mathbb {Z}^r$. In a general setting, we are interested in extension theorems for the discrete parametrized surface $S_N = \{ \mathbf {P}(\mathbf {n}) \,:\, \mathbf {n}\in [-N,N]^d \}$ lying in $\mathbb {Z}^r$. Given a sequence $a : \mathbb {Z}^d \rightarrow \mathbb {C}$ supported on $[-N,N]^d$ with $\Vert a \Vert _2 = 1$ and a weight function $\omega _d : \mathbb {Z}^d \rightarrow [0,1]$ of the form (3.1), (3.2), we define accordingly

3.3 $$\begin{aligned} F_a(\varvec{\alpha })&= \sum _{\mathbf {n}\in \mathbb {Z}^d} a(\mathbf {n}) e\big ( \mathbf {P}(\mathbf {n}) \cdot \varvec{\alpha }\big ) \quad (\varvec{\alpha }\in \mathbb {T}^r), \end{aligned}$$

3.4 $$\begin{aligned} F(\varvec{\alpha })&= \sum _{\mathbf {n}\in \mathbb {Z}^d} \omega _d(\mathbf {n}) e\big ( \mathbf {P}(\mathbf {n}) \cdot \varvec{\alpha }\big ) \quad (\varvec{\alpha }\in \mathbb {T}^r), \end{aligned}$$

which are the extension operator of our surface $S_{N}$ acting on the sequence *a* and the Fourier transform of the $\omega$-smoothed counting measure on $S_{2N}$, respectively.

For any integer $s \geqslant 1$, we define $R_{s,\mathbf {P}} : \mathbb {Z}^r \rightarrow \mathbb {C}$ at $\mathbf {u}\in \mathbb {Z}^r$ by

3.5 $$\begin{aligned} R_{s,\mathbf {P}}(\mathbf {u}) = \#\{\, \mathbf {n}_1, \dots , \mathbf {n}_s \in S \,:\, \mathbf {P}(\mathbf {n}_1) + \cdots + \mathbf {P}(\mathbf {n}_s) = \mathbf {u}\,\}. \end{aligned}$$

We have the following well-known even moment bound:

3.6 $$\begin{aligned} \Vert F_a \Vert _{2s}^{2s} \leqslant \Vert R_{s,\mathbf {P}} \Vert _\infty \Vert a \Vert _2^{2s} \leqslant \Vert F \Vert _s^s \Vert a \Vert _2^{2s}. \end{aligned}$$

This observation is occasionally useful to get $L^2 \rightarrow L^{2s}$ from bounds on moments of unweighted exponential sums or from arithmetic considerations on the number of representations by a system of polynomials. To see how (3.6) is proven, observe that

$$\begin{aligned} \Vert F_a \Vert _{2s}^{2s} = \Vert F_a^s \Vert _2^2 = \int _{\mathbb {T}^r} \Bigg | \sum _{\mathbf {u}\in \mathbb {Z}^r} \bigg ( \sum _{ \begin{array}{c} \mathbf {n}_1,\dots ,\mathbf {n}_s \in S \,: \\ \mathbf {P}(\mathbf {n}_1) + \cdots + \mathbf {P}(\mathbf {n}_s) = \mathbf {u} \end{array} } a(\mathbf {n}_1) \cdots a(\mathbf {n}_s) \bigg ) e( \varvec{\alpha }\cdot \mathbf {u}) \Bigg |^2 \mathrm {d}\varvec{\alpha }. \end{aligned}$$

By Plancherel and then by Cauchy–Schwarz, we deduce that

$$\begin{aligned} \int _{\mathbb {T}^r} |F_a|^{2s} \ \mathrm {d}m\leqslant \sum _{\mathbf {u}\in \mathbb {Z}^r} R_{s,\mathbf {P}}(\mathbf {u}) \sum _{ \begin{array}{c} \mathbf {n}_1,\dots ,\mathbf {n}_s \in S \,: \\ \mathbf {P}(\mathbf {n}_1) + \cdots + \mathbf {P}(\mathbf {n}_s) = \mathbf {u} \end{array} } |a(\mathbf {n}_1)|^2 \cdots |a(\mathbf {n}_s)|^2 \leqslant \Vert R_{s,\mathbf {P}} \Vert _{\infty } \Vert a \Vert _2^{2s}. \end{aligned}$$

The second inequality in (3.6) is obtained by orthogonality:

$$\begin{aligned} R_{s,\mathbf {P}}(\mathbf {u}) \leqslant \sum _{ \begin{array}{c} \mathbf {n}_1,\dots ,\mathbf {n}_s \in S \,: \\ \mathbf {P}(\mathbf {n}_1) + \cdots + \mathbf {P}(\mathbf {n}_s) = \mathbf {u} \end{array} } \omega _d(\mathbf {n}_1) \cdots \omega _d(\mathbf {n}_d) = \int _{\mathbb {T}^r} F(\varvec{\alpha })^s e(- \varvec{\alpha }\cdot \mathbf {u}) \mathrm {d}\varvec{\alpha }\leqslant \Vert F\Vert _s^s. \end{aligned}$$

From [3], we recall the simple technique by which one eliminates $\varepsilon$-losses in restriction estimates, using a truncated restriction estimate.

### Lemma 3.1

($\varepsilon$-removal) Suppose that

(i) $\int _{ |F_a| \geqslant C \Vert a \Vert _2 } |F_a|^p \mathrm {d}m\lesssim _\varepsilon N^{\frac{dp}{2} - K + \varepsilon } \Vert a\Vert _2^p$ for some $p \geqslant \frac{2K}{d}$ and some $C \geqslant 0$,
(ii) $\int _{ |F_a| \geqslant N^{d/2 - \zeta } \Vert a \Vert _2 } |F_a|^q \mathrm {d}m\lesssim N^{\frac{dq}{2} - K} \Vert a \Vert _2^q$ for some $q > p$ and $\zeta \in (0,\frac{d}{2})$.

If $$C \leqslant N^{d/2 - \zeta }$$, then $$\int _{ |F_a| \geqslant C \Vert a \Vert _2} |F_a|^q \mathrm {d}m\lesssim N^{\frac{dq}{2} - K} \Vert a \Vert _2^q$$.

### Remark 3.2

Unless otherwise stated, we will apply Lemma 3.1 with $C=0$ so that the set $\{ |F_a| \geqslant 0 \}$ is the entire torus $\mathbb {T}^r$ and therefore, the above bounds will apply to $\int _{\mathbb {T}^r} |F_a|^q \mathrm {d}m$.

### Proof

We may assume that $\Vert a \Vert _2 = 1$ by homogeneity. We have

$$\begin{aligned} \int _{C \leqslant |F_a| \leqslant N^{d/2 - \zeta }} |F_a|^q \mathrm {d}m\lesssim _\varepsilon (N^{\frac{d}{2} - \zeta })^{q-p} \cdot N^{\frac{dp}{2} - K + \varepsilon } \lesssim N^{\frac{dq}{2} - K + \varepsilon - (q-p)\zeta }. \end{aligned}$$

If $\varepsilon$ is chosen small enough, we obtain a bound of the desired order of magnitude, and the same bound for the integral over $\{|F_a| \geqslant N^{d/2-\zeta } \}$ is already assumed to hold. $\square$

We now discuss the discrete Tomas–Stein argument, which is the starting point of many of our later arguments. We introduce a parameter $\lambda > 0$ and define

$$\begin{aligned} E_\lambda = \{ |F_a| \geqslant \lambda \}, \qquad f = 1_{E_\lambda } \frac{F_a}{|F_a|}, \qquad g = 1_{E_\lambda }. \end{aligned}$$

This notation will be reused in later sections. Note that, by Cauchy-Schwarz in (3.3), we always have $|F_a| \leqslant CN^{d/2}$ (for instance one may take $C=3^d$), and thus we assume that the parameter $\lambda$ lies in $(0,CN^{d/2}]$.

We can view the sequences $a(\mathbf {n})$ and $\omega _d(\mathbf {n})$ in (3.3) and (3.4) as functions of $\mathbf {P}(\mathbf {n})$. Then $F = (\omega _d 1_{S_{2N}})^\wedge$ and $F_a = (a 1_{S_N})^\wedge$, and by Parseval, we have

$$\begin{aligned} \lambda |E_\lambda | \leqslant \langle f , F_a \rangle = \langle f , ( a 1_{S_N} )^\wedge \rangle = \langle \widehat{f} , a \rangle _{L^2(S_N)}. \end{aligned}$$

By Cauchy-Schwarz and under the assumption $\Vert a \Vert _2 = 1$, it follows that

$$\begin{aligned} \lambda ^2 |E_\lambda |^2 \leqslant \Vert \widehat{f} \Vert _{L^2(S_N)}^2 \leqslant \langle \widehat{f} \cdot \omega 1_{S_{2N}} , \widehat{f} \rangle . \end{aligned}$$

By another application of Parseval, we conclude that

3.7 $$\begin{aligned} \lambda ^2 |E_\lambda |^2 \leqslant \langle f *F, f \rangle \leqslant \langle g *|F| , g \rangle . \end{aligned}$$

We will use this inequality to obtain bounds of the expected order on the level sets $E_\lambda$.

Via the Hardy–Littlewood method, the kernel *F* may typically be decomposed into a main piece $F_\mathfrak {M}$ and an error term $F_\mathfrak {m}$ corresponding, respectively, to major and minor arcs, and the Tomas–Stein argument reduces matters to obtaining operator bounds for the convolution with $F_\mathfrak {M}$ and demonstrating uniform power saving on $F_\mathfrak {m}$. This strategy originated in [2, 3] and appeared for instance in [11, Section 4] and [27, Section 7] to prove $\varepsilon$-free boundedness of the extension operator applied to the curve $(x,x^2,\dots ,x^k)$; there bounds on moments of $F_\mathfrak {M}$ were used to derive operator norm bounds. The following general lemma abstracts and generalizes this approach, and it shows how to obtain a bound of the form (ii) in Lemma 3.1 from the decomposition we just described.

### Lemma 3.3

Suppose that there exists a decomposition $F = F_{\mathfrak {M}} + F_{\mathfrak {m}}$ such that

(i) $$\Vert F_{\mathfrak {M}} *f \Vert _{p} \lesssim N^{d-\frac{2K}{p}} \Vert f \Vert _{p'}$$ for some $$p > \frac{2K}{d}$$,
(ii) $\Vert F_{\mathfrak {m}} \Vert _{\infty } \lesssim N^{d(1-\tau )}$ for some $\tau \in (0,1)$.

Then $\int _{|F_a| \geqslant N^{d/2 - \zeta } \Vert a\Vert _2} |F_a|^q \mathrm {d}m\lesssim N^{\frac{dq}{2} - K} \Vert a \Vert _2^q$ holds for all $q > p$ with $\zeta = \frac{d\tau }{2}$.

### Remark 3.4

Scalar variants of this argument existed in many works of the circle method. See for instance Section 7.3 of [23]. See also [6] for a scalar version that closely resembles the one here.

### Remark 3.5

We take a moment to compare this to the Keil–Zhao device in [27], which derives its name from Theorem 4.1 of [15]. The Keil–Zhao device is Tomas’s original argument [22] (applied to discrete quadrics instead of the continuous sphere), where Keil writes out the $TT^*$ operator into an equivalent expression, before applying Tomas’s remarkable insight of decomposing the operator into various pieces and finding appropriate $L^1 \rightarrow L^\infty$ and $L^2 \rightarrow L^2$ bounds.

### Proof

We assume again that $\Vert a \Vert _2 = 1$. By (3.7),

$$\begin{aligned} \lambda ^2 |E_\lambda |^2&\leqslant \Vert f *F_{\mathfrak {M}} \Vert _p \Vert f \Vert _{p'} + \Vert f *F_{\mathfrak {m}} \Vert _\infty \Vert f \Vert _1\\&\lesssim N^{d - \frac{2K}{p}} \Vert f \Vert _{p'}^2 + \Vert F_{\mathfrak {m}} \Vert _\infty \Vert f \Vert _1^2\\&\lesssim N^{d - \frac{2K}{p}} | {E_\lambda } |^{\frac{2}{p'}} + N^{d - d\tau } | {E_\lambda } |^2. \end{aligned}$$

Therefore, for $$ \lambda \gtrsim N^{\frac{d}{2}-\frac{d\tau }{2}} $$,

$$\begin{aligned} \lambda ^2 |E_\lambda |^2 \lesssim N^{d - \frac{2K}{p}} | {E_\lambda } |^{2 - \frac{2}{p}} . \end{aligned}$$

Rearranging implies that $|E_\lambda | \lesssim \lambda ^{-p} N^{\frac{dp}{2} - K}$. The result then follows from the layer cake formula and our assumption $q > p$:

$$\begin{aligned} \int _{|F_a| \gtrsim N^{d/2 - d\tau /2}} |F_a|^q \mathrm {d}m&= q \int _{ CN^{d/2 - d\tau /2} }^{ CN^{d/2} } \lambda ^{q-1} |E_\lambda | \mathrm {d}\lambda \\&\lesssim N^{\frac{dp}{2} - K} \int _1^{CN^{d/2}} \lambda ^{q-p-1} \mathrm {d}\lambda \\&\lesssim N^{\frac{dq}{2} - K}. \end{aligned}$$

$$\square $$

The next lemma demonstrates how incorporating subcritical estimates improves the supercritical ranges.

### Lemma 3.6

Suppose that

(i) $\int _{ |F_a| \geqslant N^{d/2 - \zeta } \Vert a\Vert _2 } |F_a|^{p_1} \mathrm {d}m\lesssim N^{\frac{d p_1}{2} - K} \Vert a \Vert _2^{p_1}$ for some $p_1 > \frac{2K}{d}$ and $\zeta \in (0,\frac{d}{2})$,
(ii) $\int _{\mathbb {T}^r} |F_a|^{p_0} \mathrm {d}m\lesssim _\varepsilon N^\varepsilon \Vert a\Vert _2^{p_0}$ for some $p_0 \leqslant \frac{2K}{d}$.

Then $\int _{\mathbb {T}^r} |F_a|^{p} \mathrm {d}m\lesssim N^{\frac{dp}{2} - K} \Vert a \Vert _2^p$ holds for $p > \max [\, p_1, p_0 + \zeta ^{-1} ( K - \frac{d p_0}{2} ) ]$.

### Proof

We assume that $\Vert a\Vert _2 = 1$. The estimate of (i) at exponent $p_1$ is also valid for exponents $p \geqslant p_1$ (using the trivial bound $\Vert F_a \Vert _\infty \lesssim N^{\frac{d}{2}}$), therefore it suffices to use the second estimate to bound the tail

$$\begin{aligned} \int _{ |F_a| \leqslant N^{d/2 - \zeta } } |F_a|^p \mathrm {d}m\lesssim (N^{\frac{d}{2} - \zeta })^{p - p_0} \int _{\mathbb {T}^r} |F_a|^{p_0} \mathrm {d}m\lesssim _\varepsilon N^{\frac{dp}{2} - K} \cdot N^{K - \frac{d p_0}{2} + \varepsilon - (p - p_0) \zeta }. \end{aligned}$$

This has the desired order of magnitude under our condition on *p*. $\square$

This lemma has appeared implicitly in previous work, for example with $p_0 = 2$ in [3, Eq. (3.111)], or with $p_0 = 4$ or 6 in [12, 13]. In our work, we only use Plancherel’s theorem to exploit the subcritical estimate at $p_0 = 2$.

## Restriction estimates for *k*-th powers

In this section, we obtain truncated restriction estimates for the surface of *k*-th powers of integers, that is, for (1.6). We fix a degree $k \geqslant 3$, and for a sequence $a : \mathbb {Z}\rightarrow \mathbb {C}$ supported in [*N*] we let

4.1 $$\begin{aligned} F_a( \alpha ) = \sum _{n \in \mathbb {Z}} a(n) e( \alpha n^k ). \end{aligned}$$

In this section, we prove the first statement of Theorem 1.1, as follows.

### Theorem 4.1

Let $k \geqslant 3$ and $\tau = \max \left( 2^{1-k} , \frac{1}{k(k-1)} \right)$. For $p > 2k$, we have

$$\begin{aligned} \int _{|F_a| \geqslant N^{1/2 - \tau /2 + \varepsilon } \Vert a \Vert _2} |F_a|^p \mathrm {d}m\lesssim _\varepsilon N^{\frac{p}{2} - k} \Vert a \Vert _2^p. \end{aligned}$$

Before embarking on the proof, we derive two consequences of Theorem 4.1 mentioned in the introduction. The first consequence is the second restriction estimate of Theorem 1.1.

### Corollary 4.2

Let $k \geqslant 3$ and $\tau = \max \left( 2^{1-k} , \frac{1}{k(k-1)} \right)$. For $p > 2 + 2(k - 1)/\tau$, we have

4.2 $$\begin{aligned} \int _{\mathbb {T}} |F_a|^p \mathrm {d}m\lesssim N^{\frac{p}{2} - k} \Vert a \Vert _2^p. \end{aligned}$$

### Proof

We use Theorem 4.1 to obtain the first estimate in the assumptions of Lemma 3.6, and the trivial Plancherel estimate at $p_0 = 2$ to obtain the second one. $\square$

Secondly, we obtain the whole supercritical range of restriction estimates under Hypothesis $\text {K}$, by exploiting conjectural estimates for even exponents of lower order.

### Proof of Corollary 1.2

Assuming Hypothesis $\text {K}$ for *k*, we have $\Vert R_{k,P} \Vert _\infty \lesssim _\varepsilon N^\varepsilon$ with $P(n) = n^k$ in the notation (3.5), so that by (3.6), $\Vert F_a \Vert _{2k}^{2k} \lesssim _\varepsilon N^\varepsilon \Vert a \Vert _{2k}^{2k}$. By Theorem 4.1 and Lemma 3.1, we deduce that for $p > 2k$, the following $\varepsilon$-free estimate holds as well:

$$\begin{aligned} \int _{\mathbb {T}} |F_a|^p \ \mathrm {d}m\lesssim _p \Vert a \Vert _p^p. \end{aligned}$$

$$\square $$

We now set out to prove Theorem 4.1. We fix a sequence $a : \mathbb {Z}\rightarrow \mathbb {C}$ supported on [*N*] such that $\Vert a \Vert _2 = 1$, and a weight function $\omega$ of the form (3.1). We let

4.3 $$\begin{aligned} F( \alpha ) = \sum _{n \in \mathbb {Z}} \omega (n) e( \alpha n^k ). \end{aligned}$$

We also introduce a parameter $\lambda \in (0,N^{1/2}]$ and define

$$\begin{aligned} E_\lambda = \{ |F_a| \geqslant \lambda \}, \qquad g = 1_{E_\lambda }. \end{aligned}$$

We recall the Tomas–Stein inequality (3.7) from Sect. 3:

4.4 $$\begin{aligned} \lambda ^2 |E_\lambda |^2 \leqslant \langle g *|F|, g \rangle . \end{aligned}$$

We employ the traditional Hardy–Littlewood circle method to understand the magnitude of the exponential sum |*F*|. We set $\tau = \max \left( 2^{1-k}, \frac{1}{k(k-1)} \right)$, in accordance with the Weyl-type estimates of Appendix A we intend to use, and we fix a constant $\delta = k \tau + \varepsilon$. For a parameter $1 \leqslant Q \leqslant N^{\delta }$, we define the major and minor arcs of level *Q* by

4.5 $$\begin{aligned} \mathfrak {M}_Q(a,q)&= \Big \lbrace \alpha \in \mathbb {T}\,:\, \Big \Vert \alpha - \frac{a}{q} \Big \Vert \leqslant \frac{Q}{N^k} \Big \rbrace , \nonumber \\ \mathfrak {M}_Q&= \bigcup _{ q \leqslant Q } \bigcup _{(a,q) = 1} \mathfrak {M}_Q(a,q), \qquad \mathfrak {m}_Q = \mathbb {T}\smallsetminus \mathfrak {M}_Q. \end{aligned}$$

We take a few measures to simplify the exposition in the rest of this section. We assume implicitly that *N* is large enough with respect to *k* and $\delta$ as well as the various $\varepsilon$ quantities for the argument to work, without further indication. This is certainly possible since Theorem 4.1 with $\Vert a \Vert _2 = 1$ is trivial for *N* bounded (since $|F_a| \lesssim N^{d/2}$). With these conventions in place, we now obtain two majorants for the exponential sum *F* on minor and major arcs of level *Q*, via standard techniques from the circle method recalled in Appendix A.

### Proposition 4.3

Let $$1 \leqslant Q \leqslant N^{\delta }$$. Then

$$\begin{aligned} |F(\alpha )| \lesssim _\varepsilon {\left\{ \begin{array}{ll} N q^{\varepsilon - \frac{1}{k}} \left( 1 + N^k \left\| \alpha - \frac{a}{q} \right\| \right) ^{-\frac{1}{k}} &{} \mathrm{{if }}\; \alpha \in \mathfrak {M}_Q, \\ Q^{\varepsilon - 1/k} N &{} \mathrm{{if }}\; \alpha \in \mathfrak {m}_Q. \end{array}\right. } \end{aligned}$$

### Proof

Consider $a,q \in \mathbb {Z}$, $\beta \in \mathbb {R}$ such that $\alpha = \frac{a}{q} + \beta$, $1 \leqslant q \leqslant N^{k-1}$, $(a,q) = 1$ and $|\beta | \leqslant \frac{1}{q N^{k-1}}$. If $q \geqslant N$, then Proposition A.1 with $\theta = 0$ shows that, for $Q \leqslant N^\delta$,

$$\begin{aligned} |F(\alpha )| \lesssim _\varepsilon N^{1 - \tau + \varepsilon } \leqslant Q^{-(\tau -\varepsilon )/\delta } N \lesssim Q^{ \varepsilon ' - 1/k } N. \end{aligned}$$

Otherwise, Proposition A.2 shows that

$$\begin{aligned} |F(\alpha )| \lesssim q^{-\frac{1}{k} + \varepsilon } N ( 1 + N^k |\beta | )^{-\frac{1}{k}}. \end{aligned}$$

This gives the desired bound if $\alpha \in \mathfrak {M}_Q$, and if $\alpha \not \in \mathfrak {M}_Q$, then either $q > Q$ or $|\beta | > \frac{Q}{N^k}$, and in either case $|F(\alpha )| \lesssim _\varepsilon Q^{\varepsilon - 1/k} N$. $\square$

We define a majorant function $V_{p,Q} : \mathbb {T}\rightarrow \mathbb {C}$ by^2^

4.6 $$\begin{aligned} V_{p,Q}&= \sum _{q \leqslant Q} \ \sum _{ a \bmod q } q^{\varepsilon - p/k} \tau _{-a/q} Z_p, \end{aligned}$$

where $Z_p : \mathbb {T}\rightarrow \mathbb {C}$ is defined by

$$\begin{aligned} Z_p( \theta ) = ( 1 + N^k \Vert \theta \Vert )^{-p/k}. \end{aligned}$$

By Proposition 4.3, we have

4.7 $$\begin{aligned} |F|^p \cdot 1_{\mathfrak {M}_Q} \lesssim N^p \cdot V_{p,Q}, \qquad \Vert F 1_{\mathfrak {m}_Q} \Vert _\infty \lesssim _\varepsilon Q^{\varepsilon - \frac{1}{k}} N. \end{aligned}$$

for $1 \leqslant Q \leqslant N^\delta$. While $V_{p,Q}$ is a rather coarse majorant function, it has the advantage that its Fourier transform at nonzero frequencies can be efficiently bounded: in additive combinatorics language, it behaves pseudorandomly. This can be used in turn to obtain efficient $L^2 \rightarrow L^2$ bounds for the operator of convolution with $V_{p,Q}$. This was the approach taken by Bourgain [2] in the case of squares $k = 2$. We follow this approach and start by bounding the Fourier transform of the majorant $V_{p,Q}$ with the help of the truncated divisor functions $d(\ell ,Q) = \sum _{ n \leqslant Q \,:\, n | \ell } 1$.

### Proposition 4.4

If $p > k$, we have

4.8 $$\begin{aligned}&|\widehat{V}_{p,Q}(\ell )|&\lesssim _p N^{-k} d( \ell , Q )&(\ell \in \mathbb {Z}). \end{aligned}$$

If $p = k$, we have

4.9 $$\begin{aligned}&|\widehat{V}_{p,Q}(\ell )|&\lesssim _\varepsilon N^{\varepsilon - k}&(\ell \in \mathbb {Z}\smallsetminus \{0\}), \end{aligned}$$

4.10 $$\begin{aligned}&\widehat{V}_{p,Q}(0)&\lesssim _\varepsilon Q N^{\varepsilon - k}.&\end{aligned}$$

### Proof

By a linear change of variables, we have

$$\begin{aligned} \int _{\mathbb {T}} Z_p(\theta ) \mathrm {d}\theta \lesssim \int _0^1 ( 1 + N^k \theta )^{-p/k} \mathrm {d}\theta \lesssim N^{-k} \int _{\mathbb {R}} ( 1 + |\xi | )^{-p/k} \mathrm {d}\xi . \end{aligned}$$

By a spherical change of coordinates, we see therefore that

4.11 $$\begin{aligned} \Vert Z_p \Vert _1 \lesssim {\left\{ \begin{array}{ll} C_p N^{-k} &{} \text {if }p > k \\ C_\varepsilon N^{\varepsilon - k} &{} \text {if }p = k. \end{array}\right. } \end{aligned}$$

Recalling (4.6), this can be used to estimate $V_{p,Q}$ in $L^1$ when $p = k$:

$$\begin{aligned} \Vert V_{p,Q} \Vert _1 \leqslant \sum _{q \leqslant Q} q^{\varepsilon + 1 - p/k} \Vert Z_p \Vert _1 \lesssim _\varepsilon Q N^{\varepsilon - k}. \end{aligned}$$

Performing Fourier inversion in (4.6), we obtain also

$$\begin{aligned} \widehat{V}_{p,Q}( \ell )&= \sum _{ q \leqslant Q } q^{\varepsilon - p/k} \sum _{ a \in \mathbb {Z}_q } e_q( -a \ell ) \widehat{Z}_p(\ell ). \end{aligned}$$

By orthogonality it follows that

$$\begin{aligned} \widehat{V}_{p,Q}( \ell ) = \bigg ( \sum _{ \begin{array}{c} q \leqslant Q \\ q | \ell \end{array}} q^{\varepsilon + 1 - p/k} \bigg ) \widehat{Z}_p( \ell ). \end{aligned}$$

The sum inside the parenthesis is bounded by $N^{\varepsilon } d( \ell , Q )$ if $p = k$ and by $d( \ell , Q )$ if $p > k$ and $\varepsilon$ is small enough with respect to *p*. Using also $\Vert \widehat{Z}_p\Vert _\infty \leqslant \Vert Z_p \Vert _1$ and the estimate (4.11), this concludes the proof. $\square$

We begin by removing the minor arcs contribution to the expression (4.4), and we use $L^p$ norms to estimate the remaining piece.

### Proposition 4.5

Suppose that $\eta ^{- 2k - \varepsilon } \leqslant Q \leqslant N^{\delta }$. Then, for $p \geqslant 1$,

4.12 $$\begin{aligned} \eta ^{2p} |E_{\eta N^{1/2}}|^2 \lesssim _\varepsilon \langle V_{p,Q} , g *\widetilde{g} \rangle . \end{aligned}$$

### Proof

By (4.4), and Hölder’s inequality, it follows that

$$\begin{aligned} \lambda ^2 |E_{\lambda }|^2&\leqslant \int _\mathbb {T}|F| 1_{\mathfrak {M}_Q} d(g *\widetilde{g}) + \langle ( |F| 1_{\mathfrak {m}_Q} ) *g , g \rangle \\&\leqslant \Vert F 1_{\mathfrak {M}_Q} \Vert _{L^p(d(g *\widetilde{g}))} \cdot \Vert 1 \Vert _{L^{p'}(d(g *\widetilde{g}))} + \Vert ( |F| 1_{\mathfrak {m}_Q} ) *g \Vert _\infty \Vert g \Vert _1 \\&\leqslant \langle |F|^p 1_{\mathfrak {M}_Q} , g *\widetilde{g} \rangle ^{\frac{1}{p}} \cdot |E_\lambda |^{2 - \frac{2}{p}} + \Vert F 1_{\mathfrak {m}_Q} \Vert _\infty |E_\lambda |^2. \end{aligned}$$

Inserting the estimates of (4.7) and assuming that $\lambda ^2 \geqslant Q^{\varepsilon - 1/k} N$, we obtain

$$\begin{aligned} \lambda ^{2p} |E_{\lambda }|^2 \lesssim N^p \langle V_{p,Q} , g *\widetilde{g} \rangle . \end{aligned}$$

The proof if finished upon writing $\lambda = \eta N^{1/2}$. $\square$

We can now derive our first level set estimate, which features an $N^\varepsilon$ term.

### Proposition 4.6

Let $\zeta = \frac{\delta }{2k}$. For $p = k$, we have

4.13 $$\begin{aligned} |E_{\eta N^{1/2}}| \lesssim _\varepsilon N^{\varepsilon - k} \eta ^{-2p} \quad \text {for}\quad \eta \geqslant N^{- \zeta + \varepsilon }. \end{aligned}$$

### Proof

We assume that $\eta \geqslant N^{-\delta /2k + \varepsilon }$ and let $Q = \eta ^{- 2k - \varepsilon }$. By Proposition 4.5 with $p = k$ and Fourier inversion, it follows that

$$\begin{aligned} \eta ^{2k} |E_\lambda |^2&\leqslant \langle \widehat{V}_{k,Q} , |\widehat{g}|^2 \rangle \\&\leqslant |\widehat{V}_{k,Q}(0)| \, |\widehat{g}(0)|^2 + \Vert \widehat{V}_{k,Q} 1_{\mathbb {Z}^t \smallsetminus \{0\}} \Vert _\infty \Vert \widehat{g} \Vert _2^2. \end{aligned}$$

By Plancherel, (4.9) and (4.10), we obtain

$$\begin{aligned} \eta ^{2k} |E_\lambda |^2 \lesssim _\varepsilon Q N^{\varepsilon - k} |E_\lambda |^2 + N^{\varepsilon - k} |E_\lambda |. \end{aligned}$$

We have $\eta \geqslant N^{-\delta /2k} \geqslant N^{-1/4 + \varepsilon }$, and therefore $\eta ^{2k} \geqslant Q N^{\varepsilon - k}$, so that

$$\begin{aligned} \eta ^{2k} |E_\lambda |^2 \lesssim _\varepsilon N^{\varepsilon - k} |E_\lambda | \quad \Rightarrow \quad |E_\lambda | \lesssim _\varepsilon N^{\varepsilon - k} \eta ^{-2k}. \end{aligned}$$

$$\square $$

We now obtain a level set estimate designed to remove the $N^\varepsilon$ that arises in using Proposition 4.9 to bound the moments of $F_a$. We first introduce a technical tool to keep track of the information that the Fourier transform of *F* has support in $[N^k]$. Consider a non-negative trigonometric polynomial $\psi _N$ such that $[-N^k,N^k] \prec \widehat{\psi }_N \prec [-2N^k,2N^k]$, then $\int _\mathbb {T}\psi _N = \widehat{\psi }_N(0) = 1$. By Fourier inversion, we can see that $F = F *\psi _N$. Starting from (4.4), it is then easy to obtain the following analogue of Proposition 4.5.

### Proposition 4.7

Suppose that $\eta ^{- 2k - \varepsilon } \leqslant Q \leqslant N^{\delta }$. Then, for $p \geqslant 0$,

$$\begin{aligned} \eta ^{2p} |E_{\eta N^{1/2}}|^2 \lesssim _{\varepsilon } \langle V_{p,Q} , g *\widetilde{g} *\widetilde{\psi }_N \rangle . \end{aligned}$$

At this stage, we need to import a divisor bound used by Bourgain [2].

### Proposition 4.8

Let $B \geqslant 1$ be an integer, and suppose that $1 \leqslant Q \leqslant N^{k/B}$. Then

4.14 $$\begin{aligned} \sum _{ |\ell | \leqslant 2N^k } d( \ell , Q )^B \lesssim _{\varepsilon ,B} Q^\varepsilon N^k. \end{aligned}$$

### Proof

In the sum of (4.14), the term $\ell =0$ contributes at most $Q^B$, and by [2, eq. (4.31)] the other terms contribute at most $C_{\varepsilon ,B} Q^\varepsilon N^k$. The conclusion follows from our assumption on *Q*. $\square$

We now proceed to our $\varepsilon$-removal level set estimate.

### Proposition 4.9

Let $\nu \in (0,1]$ be a parameter. There exists a constant $c_\nu > 0$ such that, for $p > k$,

$$\begin{aligned} |E_{\eta N^{1/2}}| \lesssim _\nu N^{-k} \eta ^{-2(1+\nu )p} \qquad \text {for} \qquad \eta \geqslant N^{-c_{\nu }}. \end{aligned}$$

### Proof

We assume again that $C \eta ^{-2k - \varepsilon } \leqslant Q \leqslant N^{\delta }$, and we apply Proposition 4.7 for a fixed $p > k$. By Proposition 4.7, we have

$$\begin{aligned} \eta ^{2p} |E_{\eta N^{1/2}}|^2 \lesssim \langle V_{p,Q} *\psi _N, g *\widetilde{g} \rangle = \langle \widehat{V}_{p,Q} \, \widehat{\psi }_N, |\widehat{g}|^2 \rangle . \end{aligned}$$

Applying (4.8) and the bound $\Vert \widehat{\psi }_N \Vert _\infty \leqslant \int \psi _N = 1$, we deduce that

$$\begin{aligned} \eta ^{2p} |E_{\eta N^{1/2}}|^2&\lesssim N^{-k} \sum _{ |\ell | \leqslant 2N^k } d( \ell , Q ) |\widehat{g}(\ell )|^2. \end{aligned}$$

Let $q,q' \in [1,\infty ]$ be a dual pair of exponents to be determined later. Assuming that $q \in \mathbb {N}$ and $Q \leqslant N^{k/q}$, applications of Hölder and Proposition 4.8 furnish

$$\begin{aligned} \eta ^{2p} |E_{\eta N^{1/2}}|^2&\lesssim N^{-k} \bigg [ \sum _{ |\ell | \leqslant 2N^k } d( \ell , Q )^q \bigg ]^{\frac{1}{q}} \bigg [ \sum _{ |\ell | \leqslant 2N^k } |\widehat{g}(\ell )|^{2q'} \bigg ]^{\frac{1}{q'}} \\&\lesssim _{q,\varepsilon } N^{-k} ( Q^\varepsilon N^k )^{1/q} \Vert \widehat{g} \Vert _\infty ^{2 - 2/q'} ( \Vert \widehat{g} \Vert _2^2 )^{1/q'} \\&\leqslant N^{ - k/q'} Q^{\varepsilon /q} |E_{\eta N^{1/2}}|^{2 - 1/q'}. \end{aligned}$$

Rearranging terms in the above, we find that

$$\begin{aligned} |E_{\eta N^{1/2}}| \lesssim _{q,\varepsilon } N^{-k} Q^{\varepsilon q'/q} \eta ^{-2 q' p}. \end{aligned}$$

Choose finally $Q = C \eta ^{- 2k - 2\varepsilon }$ and *q* a large enough integer so that $q' < 1 + \nu$, and note that we obtain the desired bound for $\varepsilon$ small enough. The condition $Q \leqslant N^{k/q}$ is satisfied for $\eta \geqslant N^{-c_\nu }$ with a certain $c_\nu > 0$. $\square$

We now prove Theorem 4.1 (with the rescaling $\Vert a \Vert _2 = 1$) by integrating the previous level set estimates.

### Proposition 4.10

Let $\zeta = \frac{\delta }{2k}$. We have

4.15 $$\begin{aligned} \int _{|F_a| \geqslant N^{1/2 - \zeta + \varepsilon }} |F_a|^p \ \mathrm {d}m\lesssim _\varepsilon N^{\frac{p}{2} - k + \varepsilon } \quad \text {for}\quad p \geqslant 2k. \end{aligned}$$

There exists $c_\nu > 0$ such that, for $p > 2k$,

4.16 $$\begin{aligned} \int _{|F_a| \geqslant N^{1/2-c_\nu }} |F_a|^p \ \mathrm {d}m\lesssim _p N^{\frac{p}{2} - k} \quad \text {for}\quad p > 2k. \end{aligned}$$

### Proof

By the layer cake formula and Proposition 4.6, we obtain

$$\begin{aligned} \int _{ |F_a| \geqslant N^{1/2 - \zeta + \varepsilon } } |F_a|^p \ \mathrm {d}m&\asymp N^{p/2} \int _{N^{- \zeta + \varepsilon }}^1 \eta ^{p-1} |E_{\eta N^{1/2}}| \mathrm {d}\eta \\&\lesssim _\varepsilon N^{\frac{p}{2} - k + \varepsilon } \int _{N^{- \zeta + \varepsilon }}^1 \eta ^{p - 2k - 1} \mathrm {d}\eta \\&\lesssim _\varepsilon N^{\frac{p}{2} - k + \varepsilon }, \end{aligned}$$

where $p \geqslant 2k$ ensured that the $\eta$-integral is $\lesssim \log N$.

The second estimate is obtained similarly, by invoking Proposition 4.9 in place of Proposition 4.6. $\square$

### Proof of Theorem 4.1

Remember that $\delta > k\tau$ was arbitrary, and therefore the parameter $\zeta$ in Proposition 4.10 can be given a value arbitrarily close to $\frac{\tau }{2}$. Fixing $\zeta$ as such and taking $C := N^{1/2-\zeta }$ along with $0<c_v<\zeta$, we can now use (4.16) in Lemma 3.1, to remove the $N^\varepsilon$ factor in (4.15) for $p > 2k$ which is the desired estimate of Theorem 4.1. $\square$

## Extending the moment method

The method of the previous section extends to many surfaces, due to its reliance on little number-theoretic information. However, it does not seem to produce truncated restriction estimates in the complete supercritical range for many interesting cases, and therefore we only sketch this class of results.

Fix $t \geqslant 1$ and a tuple of integers $\mathbf {k}\in \mathbb {Z}^t$ with $1 \leqslant k_1< \cdots < k_t$. We consider the monomial curve

$$\begin{aligned} S = \{ (n^{k_1},\dots ,n^{k_t}) \,:\, n \in [N] \}. \end{aligned}$$

Define also the maximal degree $k = k_t$ and the total degree $K = k_1 + \cdots + k_t$. For a sequence $a : \mathbb {Z}\rightarrow \mathbb {C}$ supported on [*N*] define the following exponential sums associated to *S*:

$$\begin{aligned}&F( \varvec{\alpha })&= \sum _{n \in [N]} e( \alpha _1 n^{k_{t_1}} + \cdots + \alpha _t n^{k_t} )&(\varvec{\alpha }\in \mathbb {T}^t),\\&F_a( \varvec{\alpha })&= \sum _{n \in \mathbb {Z}} a(n) e( \alpha _1 n^{k_{t_1}} + \cdots + \alpha _t n^{k_t} )&(\varvec{\alpha }\in \mathbb {T}^t). \end{aligned}$$

It can be checked that the method of Sect. 4 yields truncated restriction exponents in the range $p > 2kt$, which is quite far from the full supercritical range $p > 2(k_1 + \cdots + k_t)$ for large values of *t*. It turns out to be more useful to use a different majorant in that situation. We only describe the main steps of this variant since it was already derived in the case $\mathbf {k}= (1,\dots ,k)$ in previous work (see [11, Section 4], [27, Section 7]). By the circle method, one can obtain a decomposition of the form $F = F_\mathfrak {M}+ F_\mathfrak {m}$ with

5.1 $$\begin{aligned} \Vert F_\mathfrak {M}\Vert _p^p \lesssim \mathfrak {S}_p \cdot \mathfrak {J}_p \cdot N^{p-K}, \qquad \Vert F_\mathfrak {m}\Vert _\infty \lesssim _\varepsilon N^{1-\tau +\varepsilon }, \end{aligned}$$

where $\mathfrak {S}_p$ and $\mathfrak {J}_p$ are, respectively, the singular series and the singular integral defined by

$$\begin{aligned} \mathfrak {S}_p&= \sum _{q \geqslant 1 } \sum _{(\mathbf {a},q) = 1} \bigg | \sum _{(\mathbf {a},q) = 1} e_q( a_1 u^{k_1} + \cdots + a_t u^{k_t} ) \bigg |^p, \\ \mathfrak {J}_p&= \int _{\mathbb {R}^t} \bigg | \int _{\mathbb {R}} e( \xi _1 x^{k_1} + \cdots + \xi _t x^{k_t} ) \mathrm {d}x\bigg | \mathrm {d}\varvec{\xi }. \end{aligned}$$

It is known from classical work of Hua [14] and Arkhipov–Chubarikov*-Karatsuba [1, Theorems 1.3, 1.4, 2.4, 2.5], that when $\mathbf {k}= (1,\dots ,k)$, $\mathfrak {J}_p < \infty$ for $p > K + 1$ and $\mathfrak {S}_p < \infty$ for $p > K+2$, while when $\mathbf {k}\ne (1,\dots ,k)$ and $k \geqslant 4$, $\mathfrak {J}_p < \infty$ for $p > K$ and $\mathfrak {S}_p < \infty$ for $p > K + 1$. Via Lemma 3.3, and writing $\rho = \tau /2$, this gives

$$\begin{aligned} \int _{|F_a| \geqslant N^{1/2 - \rho }} |F_a|^q \mathrm {d}m\lesssim N^{\frac{q}{2} - K} \quad \text {for }q > 2K + 2 \end{aligned}$$

if $\mathbf {k}\ne (1,\dots ,k)$ and $k \geqslant 4$, and

$$\begin{aligned} \int _{|F_a| \geqslant N^{1/2 - \rho }} |F_a|^q \mathrm {d}m\lesssim N^{\frac{q}{2} - K} \quad \text {for }q > 2K + 4 \end{aligned}$$

if $\mathbf {k}= (1,\dots ,k)$. (This last estimate is the one that was already obtained in [11] and [27]). Note that the above ranges of exponent miss the conjectured ones by two or four variables only.

## Arc mollifiers

This section serves to introduce a technical tool, borrowed from Bourgain [3, Section 3] and used in the proof of Theorems 1.3 and 1.4. It consists of a collection of multipliers in the frequency variable $\alpha \in \mathbb {T}$, which serves as a partition of unity adapted to the major arcs, that is, the collection of small neighborhoods of rationals with small denominator. We recall the natural bounds on these multipliers and their Fourier transform. Throughout the section we fix an integer $k \geqslant 3$, which corresponds to the degree *k* of the *k*-paraboloid in Sects. 7 and 8.

We fix a smooth bump function $\kappa$ with $[-1,1] \prec \kappa \prec [-2,2]$. Let $\widetilde{N} = 2^{\lfloor \log _2 N \rfloor }$, and for every integer $0 \leqslant s \leqslant \lfloor \log _2 N \rfloor$ define

6.1 $$\begin{aligned} \phi ^{(s)} :={\left\{ \begin{array}{ll} \kappa ( 2^s N^{k-1} \,\cdot \, ) - \kappa ( 2^{s+1} N^{k-1} \,\cdot \, ) &{} \text {if }1 \leqslant 2^s < \widetilde{N},\\ \kappa ( 2^{s} N^{k-1} \, \cdot \,) &{} \text {if }2^s = \widetilde{N}. \end{array}\right. } \end{aligned}$$

Note that we have6.2More importantly, for every dyadic integer $1 \leqslant Q \leqslant N$, we have

6.3 $$\begin{aligned} \sum _{Q \leqslant 2^s \leqslant N} \phi ^{(s)} = \kappa ( Q N^{k-1} \,\cdot \,). \end{aligned}$$

We let $N_1 = c_1 N$, for a small constant $c_1 \in (0,1]$. It is then easy to check that the intervals

6.4 $$\begin{aligned} \frac{a}{q} + \bigg [-\frac{2}{QN^{k-1}} , \frac{2}{QN^{k-1}} \bigg ], \quad 1 \leqslant a \leqslant q,\ q \sim Q,\ 1\leqslant Q \leqslant N_1 \end{aligned}$$

are all disjoint. For a dyadic integer *Q* and an integer $0 \leqslant s \leqslant \log _2 N$, we define the arc mollifier

6.5 $$\begin{aligned} \Phi _{Q,s} = \sum _{\begin{array}{c} (a,q) = 1 \\ q \sim Q \end{array}} \tau _{-a/q} \phi ^{(s)}, \end{aligned}$$

so that, by (6.2) and disjointness,6.6We finally define

6.7 $$\begin{aligned} \lambda = \sum _{Q \leqslant N_1} \sum _{Q \leqslant 2^{s} \leqslant N} \Phi _{Q,s}, \qquad \rho = 1 - \lambda . \end{aligned}$$

### Proposition 6.1

We have $0 \leqslant \lambda , \rho \leqslant 1$ and

$$\begin{aligned} \lambda = 1,\ \rho = 0 \quad \text {on}\quad \bigsqcup _{Q \leqslant N_1} \bigsqcup _{\begin{array}{c} (a,q) = 1 \\ q \sim Q \end{array}} \bigg ( \frac{a}{q} + \bigg [-\frac{1}{QN^{k-1}}, \frac{1}{QN^{k-1}} \bigg ] \bigg ). \end{aligned}$$

### Proof

By (6.3), we can rewrite $\lambda$ as

$$\begin{aligned} \lambda = \sum _{Q \leqslant N_1} \sum _{\begin{array}{c} (a,q) = 1 \\ q \sim Q \end{array}} \tau _{-a/q} \bigg ( \sum _{Q \leqslant 2^s \leqslant N} \phi ^{(s)} \bigg ) = \sum _{Q \leqslant N_1} \sum _{\begin{array}{c} (a,q) = 1 \\ q \sim Q \end{array}} \tau _{-a/q} \kappa (QN^{k-1} \,\cdot \,). \end{aligned}$$

The proposition follows since we assumed that $[-1,1] \prec \eta \prec [-2,2]$ and the intervals (6.4) are disjoint. $\square$

At this stage we define the fundamental domain $\mathcal {U}= ( \frac{1}{2N_1} , 1 + \frac{1}{2N_1}]$, and we note that when *N* is large, then for every $1 \leqslant a \leqslant q \leqslant Q \leqslant N_1$, we have

$$\begin{aligned} \frac{a}{q} + \bigg [ - \frac{2}{QN^{k-1}} , \frac{2}{QN^{k-1}} \bigg ] \subset \overset{\circ }{\mathcal {U}} \end{aligned}$$

Therefore for $1 \leqslant Q \leqslant 2^s \leqslant N$, the functions $\phi ^{(s)}$, $\Phi _{Q,s}$ and $\lambda$ are supported on $\overset{\circ }{\mathcal {U}}$, the interior of ${\mathcal {U}}$, and they may be viewed as smooth functions over the torus $\mathbb {T}$, by 1-periodization from the interval $\mathcal {U}$. We will view $\Phi _{Q,s}$ alternatively as a smooth function on the torus $\mathbb {T}$ or on the real line, but note that for an integer *n*, $\widehat{\Phi _{Q,s}}(n)$ has the same definition under both points of view.

For $n \in \mathbb {Z}$ and an integer $Q \geqslant 1$ we define

$$\begin{aligned} d(n,Q) = \sum _{\begin{array}{c} \ 1 \leqslant d \leqslant Q \,: \\ d|n \end{array}} 1. \end{aligned}$$

The following useful lemma is due to Bourgain [3]. We include the short proof for completeness.

### Lemma 6.2

Let $\delta _x$ be the Dirac function at *x*. Then

$$\begin{aligned}&\widehat{ \sum _{\begin{array}{c} (a,q) = 1 \\ q \sim Q \end{array}} \delta _{a/q} }(n) \lesssim Q \cdot d(n,2Q)&(n \in \mathbb {Z}). \end{aligned}$$

### Proof

We note that $\sum _{(a,q) = 1} \widehat{\delta _{a/q}}(n) = \sum _{(a,q) = 1} e_q( a n ) = c_q(n)$ is a Ramanujan sum. By a well-known convolution identity [19, Theorem 4.1], we have then

$$\begin{aligned} \bigg | \sum _{q \sim Q} c_q(n) \bigg | = \bigg | \sum _{q \sim Q} \sum _{d | (q,n)} d \mu (q/d) \bigg | \leqslant \sum _{\begin{array}{c} d | n \\ d \leqslant 2Q \end{array}} \ d \ \sum _{\begin{array}{c} q \sim Q \\ d | q \end{array}} 1 \lesssim Q \sum _{\begin{array}{c} d | n \\ d \leqslant 2Q \end{array}} 1, \end{aligned}$$

and the leftmost term above is exactly $| \sum _{q \sim Q} \sum _{(a,q)=1} \widehat{\delta _{a/q}}(n) |$. $\square$

### Proposition 6.3

We have

6.8 $$\begin{aligned}&\int \Phi _{Q,s} \mathrm {d}m&\lesssim \frac{Q^2}{2^s N^{k-1}},&\end{aligned}$$

6.9 $$\begin{aligned}&\widehat{\Phi _{Q,s}}(n)&\lesssim \frac{Q}{2^s N^{k-1}} d(n,2Q)&(n \in \mathbb {Z}) \end{aligned}$$

### Proof

Let $\gamma ^{(s)} = \kappa - \kappa (2 \,\cdot \,)$ for $0 \leqslant s < \lfloor \log _2 N \rfloor$ and $\gamma ^{(s)} = \kappa$ when $s = \lfloor \log _2 N \rfloor$. By (6.1) and (6.5), we can write

$$\begin{aligned} \Phi _{Q,s} = \sum _{\begin{array}{c} (a,q)=1 \\ q \sim Q \end{array}} \tau _{-a/q} \gamma ^{(s)}(2^s N^{k-1} \,\cdot \, ) = \bigg ( \sum _{\begin{array}{c} (a,q)=1 \\ q \sim Q \end{array}} \delta _{a/q} \bigg ) *\gamma ^{(s)}(2^s N^{k-1} \,\cdot \, ). \end{aligned}$$

From Lemma 6.2, we deduce the pointwise bound

$$\begin{aligned} |\widehat{\Phi _{Q,s}}(n)| = \bigg | \widehat{\sum _{\begin{array}{c} (a,q)=1 \\ q \sim Q \end{array}} \delta _{a/q}}(n) \cdot \frac{1}{ 2^s N^{k-1} } \widehat{\gamma ^{(s)}}\Big ( \frac{n}{2^s N^{k-1}} \Big ) \bigg | \lesssim \frac{Q}{ 2^s N^{k-1} } d(n,2Q), \end{aligned}$$

which is uniform in $n \in \mathbb {Z}$. When $n = 0$ the left-hand side is $\int \Phi _{Q,s} \mathrm {d}m$. $\square$

### Proposition 6.4

For every $\varepsilon > 0$ and $A > 0$, we have

6.10 $$\begin{aligned} \int \rho \,\mathrm {d}m&\asymp 1, \end{aligned}$$

6.11 $$\begin{aligned} \widehat{\rho }(n)&\lesssim _{\varepsilon ,A} \frac{1}{N^{k-1-\varepsilon }} \quad \text {for }0 < |n| \leqslant A N^A. \end{aligned}$$

### Proof

Since *Q* is dyadic, it follows from (6.7) and (6.8) that

$$\begin{aligned} \int \rho \,\mathrm {d}m&= 1 - O\bigg ( \sum _{Q \leqslant N_1} \sum _{Q \leqslant 2^s \leqslant N} \frac{Q^2}{2^s N^{k-1}} \bigg ) \\&= 1 - O\bigg ( \frac{1}{N^{k-1}} \sum _{Q \leqslant N_1} Q \bigg ) \\&= 1 - O\Big ( \frac{N_1}{N^{k-1}} \Big ). \end{aligned}$$

Since we have chosen $N_1 = c_0 N$ with $c_1$ small enough, we have $\int \rho \mathrm {d}m\asymp 1$ as desired. The bound on $\widehat{\rho }$ is derived from (6.9) in a similar fashion, using also the standard divisor bound $d(n,Q) \leqslant d(n) \lesssim _\varepsilon n^\varepsilon$. $\square$

## Restriction estimates for *k*-paraboloids of arbitrary dimension

In this section, we obtain truncated restriction estimates for the surface (1.7), for an arbitrary dimension $d \geqslant 1$ and degree $k \geqslant 3$. For simplicity, we write $|\mathbf {x}|_k = (x_1^k + \dots + x_d^k)^{1/k}$ for vectors $\mathbf {x}\in \mathbb {R}^d$; this quantity may be negative when *k* is odd. Note that the system of polynomials $\mathbf {P}= (\mathbf {x},|\mathbf {x}|_k^k)$ has total degree $K = d+k$, and therefore the critical restriction exponent is $p_{d,k} = \frac{2(d+k)}{d}$ for the surface (1.7). For a sequence $a : \mathbb {Z}^d \rightarrow \mathbb {C}$ supported on $[-N,N]^d$, we let

7.1 $$\begin{aligned} F_a(\alpha ,\varvec{\theta })&= \sum _{\mathbf {n}\in \mathbb {Z}^d} a(\mathbf {n}) e( \alpha |\mathbf {n}|_k^k + \varvec{\theta }\cdot \mathbf {n})&(\alpha \in \mathbb {T}, \varvec{\theta }\in \mathbb {T}^d). \end{aligned}$$

The following estimate, a slightly more precise version of the first statement in Theorem 1.3, is the main result of this section. Note that we miss the complete supercritical range by a term of size $\frac{2k}{d}$, but we obtain a uniform result for all dimensions *d* and degrees *k*.

### Theorem 7.1

Suppose that $d \geqslant 1$ and $k \geqslant 3$, and let $\tau = \max (2^{1-k},\frac{1}{k(k-1)})$. For every $p > \frac{2(d+k)}{d} + \frac{2k}{d}$ and $\varepsilon > 0$, we have

$$\begin{aligned} \int _{|F_a| \geqslant N^{d/2 - d\tau /2 + \varepsilon } \Vert a\Vert _2 } |F_a|^p \mathrm {d}m\lesssim _{p,\varepsilon } N^{\frac{dp}{2} - (d + k)} \Vert a \Vert _2^p. \end{aligned}$$

We record below the corresponding restriction estimate that can be obtained by bounding the tail of the integral.

### Corollary 7.2

Suppose that $d \geqslant 1$ and $k \geqslant 3$, and let $\tau = \max (2^{1-k},\frac{1}{k(k-1)})$. The restriction estimate $\int |F_a|^p \mathrm {d}m\lesssim N^{\frac{dp}{2} - (d + k)} \Vert a\Vert _2^p$ holds for $p > 2 + \frac{2k}{d\tau }$.

### Proof

We invoke Lemma 3.6. The first assumption is verified with $\zeta \leftarrow \frac{d\tau }{2} - \varepsilon$ for any $p_1 > \frac{2(d + 2k)}{d}$ by Theorem 7.1 and the second is verified for $p_0 = 2$ by Plancherel. Since $2 + \frac{2k}{d\tau } \geqslant \frac{2(d+2k)}{d}$, we obtain a range of exponents $p > 2 + \frac{2k}{d\tau }$. $\square$

Our argument will make use of Lemma 3.3, whose philosophy borrows from the circle method the paradigm of major arc and minor arc estimates. As such we will split our convolution kernel *F*, defined in (7.2) below, into major arc pieces and a minor arc piece. On the minor arc piece we will only need some power savings on the trivial bound. We decompose the major arc pieces in a fashion similar to [3], but simpler, and we use the Tomas–Stein method to obtain decent estimates.

We introduce some notation before turning to our proof. We fix integers $d \geqslant 1$ and $k \geqslant 3$ throughout, on which every implicit or explicit constant throughout is allowed depend. The letter *Q* will always denote an integer of the form $2^r$ with $r \geqslant 0$. We also fix weight functions $\omega$ and $\omega _d$ of the form (3.1) and (3.2), and we define the exponential sums

7.2 $$\begin{aligned} F( \alpha , \varvec{\theta })&= \sum _{ \mathbf {n}\in \mathbb {Z}^d } \omega _d(\mathbf {n}) e( \alpha |\mathbf {n}|_k^k + \varvec{\theta }\cdot \mathbf {n})&(\alpha \in \mathbb {T},\ \varvec{\theta }\in \mathbb {T}^d), \end{aligned}$$

7.3 $$\begin{aligned} T( \alpha , \theta )&= \sum _{ n \in \mathbb {Z}} \omega (n) e( \alpha n^k + \theta n )&(\alpha \in \mathbb {T},\ \theta \in \mathbb {T}), \end{aligned}$$

which may be viewed as Fourier transforms of smoothed surface measures on $\{ (|\mathbf {n}|_k^k,\mathbf {n}) \,:\, \mathbf {n}\in [-2N,2N]^d \}$, respectively, for general *d* and for $d = 1$.

Note that the sum over $\mathbf {n}\in \mathbb {Z}^d$ in (7.2) splits and we have

7.4 $$\begin{aligned} F( \alpha , \varvec{\theta }) = \prod _{i=1}^d T( \alpha , \theta _i ). \end{aligned}$$

Another useful observation is that

7.5 $$\begin{aligned} {{\mathrm{Supp}}}(\widehat{F}) \subset [-d(2N)^k,d(2N)^k] \times [-2N,2N]^d. \end{aligned}$$

For each dyadic integer *Q* and integer $s \geqslant 0$ such that $1 \leqslant Q \leqslant 2^s$, we define a piece of our original exponential sum by

7.6 $$\begin{aligned} F^{Q,s}(\alpha ,\varvec{\theta }) = \Phi _{Q,s}(\alpha ) \cdot F(\alpha ,\varvec{\theta }) . \end{aligned}$$

Recall that the weight $\Phi _{Q,s}$ is essentially a mollified indicator of the $\frac{1}{2^sN}$-neighborhood of the set of rationals with denominator of size *Q*.

We now define the piece $F_\mathfrak {M}$ of our exponential sum corresponding to the union of all major arcs, and the piece $F_\mathfrak {m}$ corresponding to the minor arcs, by

7.7 $$\begin{aligned} F_{\mathfrak {M}} = \sum _{Q \leqslant N_1} \, \sum _{Q \leqslant 2^s \leqslant N} F^{Q,s}, \qquad F_{\mathfrak {m}} = F - F_{\mathfrak {M}}. \end{aligned}$$

Recalling the decomposition (6.7), this means that

7.8 $$\begin{aligned} F_\mathfrak {m}(\alpha ,\varvec{\theta }) = \rho (\alpha ) F(\alpha ,\varvec{\theta }). \end{aligned}$$

We fix a Weyl exponent $\tau = \max \left( 2^{1-k}, \frac{1}{k(k-1)}\right)$. The minor arc estimates of Appendix A translate into the following statement.

### Proposition 7.3

Uniformly in $\alpha \in \mathbb {T}$, $\varvec{\theta }\in \mathbb {T}^d$, we have

$$\begin{aligned} \rho (\alpha ) \ne 0 \quad \Rightarrow \quad |F(\alpha ,\varvec{\theta })|&\lesssim _\varepsilon N^{d - d\tau + \varepsilon }. \end{aligned}$$

### Proof

Consider $\alpha \in \mathcal {U}$ such that $\rho (\alpha ) \ne 0$. Take $1 \leqslant a \leqslant q \leqslant N^{k-1}$ such that $(a,q) = 1$ and $|\alpha - a/q| \leqslant 1/qN^{k-1}$. It follows from Proposition 6.1 that $q > N_1$, for else there exists a dyadic integer *Q* such that $q \sim Q \Rightarrow Q \leqslant N_1$ and $|\alpha - a/q| \leqslant 1/QN^{k-1}$, a contradiction. Therefore we have $N \lesssim q \leqslant N^{k-1}$ and we may apply the bound of Proposition A.1 to each Weyl sum in the product (7.4). $\square$

By (7.8), we have the following immediate corollary.

### Corollary 7.4

We have

7.9 $$\begin{aligned} \Vert F_{\mathfrak {m}} \Vert _\infty \lesssim _{\varepsilon } N^{d - d\tau + \varepsilon } . \end{aligned}$$

We can derive a bound on the piece $F^{Q,s}$ of the exponential sum by appealing to major arc bounds.

### Proposition 7.5

We have, uniformly for $Q \leqslant 2^s \leqslant N$,

$$\begin{aligned} \Vert F^{Q,s} \Vert _\infty \lesssim _\varepsilon Q^\varepsilon \bigg ( \frac{2^s}{Q} \bigg )^{\frac{d}{k}} N^{d(1-\frac{1}{k})}. \end{aligned}$$

### Proof

Consider $\alpha \in \mathcal {U}$. By (7.4) and (7.6), we have

$$\begin{aligned} |F^{Q,s}(\alpha ,\varvec{\theta })| \leqslant \Phi _{Q,s}(\alpha ) \prod _{j=1}^d |T(\alpha ,\theta _i)|. \end{aligned}$$

If $\Phi _{Q,s}(\alpha ) \ne 0$, then it follows from (6.6) that there exist $1 \leqslant a \leqslant q$ with $(a,q) = 1$, $q \sim Q$ such that $|\alpha - \frac{a}{q}| \asymp \frac{1}{2^s N^{k-1}}$ if $2^s < \widetilde{N}$, or $|\alpha - \frac{a}{q}| \lesssim \frac{1}{2^s N^{k-1}}$ if $2^s = \widetilde{N}$. By Proposition A.2, we have in both cases

$$\begin{aligned} |F^{Q,s}(\alpha ,\varvec{\theta })| \lesssim _\varepsilon Q^{-\frac{d}{k} + \varepsilon } (2^s N^{k-1} )^{\frac{d}{k}}. \end{aligned}$$

$$\square $$

### Proposition 7.6

We have

$$\begin{aligned} \Vert \widehat{F^{Q,s}} \Vert _\infty \lesssim \frac{Q^2}{2^s N^{k-1}}. \end{aligned}$$

### Proof

For any $(m,\varvec{\ell }) \in \mathbb {Z}^{d+1}$, we have

$$\begin{aligned} \widehat{F^{Q,s}}(m,\varvec{\ell })&= \int _{\mathbb {T}^{d+1}} \Phi _{Q,s}(\alpha ) F(\alpha ,\varvec{\theta }) e( - \alpha m - \varvec{\theta }\cdot \varvec{\ell }\, ) \mathrm {d}\alpha \mathrm {d}\varvec{\theta }\\&= \sum _{\mathbf {n}\in \mathbb {Z}^d} \omega _d(\mathbf {n}) \int _{\mathbb {T}^{d+1}} \Phi _{Q,s}(\alpha ) e\big ( \alpha ( |\mathbf {n}|_k^k - m ) + \varvec{\theta }\cdot ( \mathbf {n}- \varvec{\ell }) \big ) \mathrm {d}\alpha \mathrm {d}\varvec{\theta }\\&= \omega _d(\varvec{\ell }) \widehat{\Phi }_{Q,s}( m - |\varvec{\ell }|_k^k ). \end{aligned}$$

The result now follows from (6.8) and the trivial bound $\Vert \widehat{\Phi }_{Q,s} \Vert _\infty \leqslant \Vert \Phi _{Q,s} \Vert _1$. $\square$

From the previous physical and Fourier-side estimates on a major arc piece $F^{Q,s}$, we immediately deduce $L^1 \rightarrow L^\infty$ and $L^2 \rightarrow L^2$ estimates for the operator of convolution with this piece.

### Proposition 7.7

Uniformly for $Q \leqslant 2^s \leqslant N$, we have

7.10 $$\begin{aligned} \Vert F^{Q,s} *f \Vert _\infty&\lesssim _{\varepsilon } Q^\varepsilon \Big ( \frac{2^s}{Q} \Big )^{\frac{d}{k}} N^{d(1-\frac{1}{k})} \Vert f \Vert _1, \end{aligned}$$

7.11 $$\begin{aligned} \Vert F^{Q,s} *f \Vert _2&\lesssim _\varepsilon \frac{Q^2}{2^s N^{k-1}} \Vert f \Vert _2. \end{aligned}$$

### Proof

First note that for any bounded function $W : \mathbb {T}^{d+1} \rightarrow \mathbb {C}$, we have

$$\begin{aligned} \Vert W *f \Vert _\infty \leqslant \Vert W \Vert _\infty \Vert f \Vert _1, \qquad \Vert W *f \Vert _2 = \Vert \widehat{W} \widehat{f} \Vert _2 \leqslant \Vert \widehat{W} \Vert _\infty \Vert f \Vert _2. \end{aligned}$$

Applying these two inequalities to $W = F^{Q,s}$, and inserting the estimates of Propositions 7.5 and 7.6, we obtain the desired bounds. $\square$

Interpolation between the previous convolution estimates gives the following result.

### Proposition 7.8

Let $p'_0 = \frac{2(k+d)}{d}$ and $p \in (1,2]$. Uniformly for $Q \leqslant 2^s \leqslant N$, we have

7.12 $$\begin{aligned} \Vert F^{Q,s} *f \Vert _{p'} \lesssim _{\in , \rho } Q^{\frac{2}{p'} + \varepsilon } \Big [ \Big ( \frac{2^s}{Q} \Big )^\frac{d}{k} N^{d(1-\frac{1}{k})}\Big ]^{1 - \frac{p'_0}{p'}} \Vert f \Vert _p. \end{aligned}$$

### Proof

Fix parameters $p \in (1,2]$ and $\theta \in (0,1]$ such that

7.13 $$\begin{aligned} \frac{1}{p'} = \frac{1-\theta }{\infty } + \frac{\theta }{2}, \qquad \frac{1}{p} = \frac{1-\theta }{1} + \frac{\theta }{2}. \end{aligned}$$

By interpolation between the estimates of Proposition 7.7, we obtain

$$\begin{aligned} \Vert F^{Q,s} *f \Vert _{p'}&\lesssim _{\in ,\rho } Q^\varepsilon \Big ( \frac{2^s}{Q} \Big )^{(1-\theta ) \frac{d}{k} } N^{d(1-\frac{1}{k})(1-\theta )} \cdot \Big ( \frac{Q}{2^s} \Big )^\theta \Big ( \frac{Q}{N^{k-1}} \Big )^\theta \cdot \Vert f \Vert _p \\&\lesssim Q^{\theta +\varepsilon } \cdot \Big ( \frac{2^s}{Q} \Big )^{ \frac{d}{k} - \frac{d}{k}(1+\frac{k}{d})\theta } \cdot N^{d(1-\frac{1}{k}) - \theta (d(1-\frac{1}{k}) + k(1-\frac{1}{k}) ) } \cdot \Vert f \Vert _p \\&\lesssim Q^{\theta + \varepsilon } \cdot \Big [ \Big ( \frac{2^s}{Q} \Big )^{\frac{d}{k}} N^{ d(1-\frac{1}{k})} \Big ]^{1 - \frac{k+d}{d} \theta } \cdot \Vert f \Vert _p. \end{aligned}$$

Since $\theta = \frac{2}{p'}$, we see that $1 - \frac{k+d}{d} \theta = 1 - \frac{p'_0}{p'}$, which yields the desired estimate. $\square$

We need to sum this up over the major arcs.

### Proposition 7.9

If $$ p' > \frac{2(d+k) + 2k}{d} $$, then

7.14 $$\begin{aligned} \Vert F_\mathfrak {M}*f \Vert _{p'} \lesssim N^{d - \frac{2(d+k)}{p'}} \Vert f \Vert _p. \end{aligned}$$

### Proof

When $p' > p'_0$, Proposition 7.8 and the triangle inequality yield

$$\begin{aligned} \Vert F_\mathfrak {M}*f \Vert _{p'}&\leqslant \sum _{Q \leqslant N} \sum _{Q \leqslant 2^s \leqslant N_1} \Vert F^{Q,s} *f \Vert _{p'} \\&\lesssim \sum _{Q \leqslant N} \sum _{Q \leqslant 2^s \leqslant N_1} Q^{\frac{2}{p'} + \varepsilon } \Big ( \frac{2^s}{Q} \Big )^{ \frac{d}{k} (1 - \frac{p'_0}{p'}) } N^{ d(1-\frac{1}{k}) (1 - \frac{p'_0}{p'}) } \; \Vert f \Vert _{p} \\&\leqslant \sum _{Q \leqslant N} Q^{\frac{2}{p'} - \frac{d}{k} ( 1 - \frac{p'_0}{p'} ) + \varepsilon } N^{ d (1 - \frac{p'_0}{p'}) } \; \Vert f \Vert _{p} . \end{aligned}$$

The sum over the dyadic *Q* is *O*(1) for $(2 + \frac{d p'_0}{k}) \frac{1}{p'} < \frac{d}{k}$, which gives the range stated in the proposition. $\square$

### Proof of Theorem 7.1

We have a decomposition $F = F_\mathfrak {M}+ F_\mathfrak {m}$ which satisfies the estimates of Propositions 7.4 and 7.9. The result now follows from Lemma 3.3, recalling that $\tau = \max \left( 2^{1-k}, \frac{1}{k(k-1)} \right)$. $\square$

## Restriction estimates for *k*-paraboloids of low dimension

In this section, we pursue the study of *k*-paraboloids of the form (1.7) initiated in Sect. 7, but we aim at obtaining results valid in the complete supercritical range of exponents $p > \frac{2(d + k)}{d}$ instead, under a constraint on the dimension *d*. The following is the main result of this section, which corresponds to Theorem 1.4. Here $F_a$ is defined by (7.1) as before.

### Theorem 8.1

Suppose that $d \geqslant 1$, $k \geqslant 3$ and let $\tau = \max \left( 2^{1-k}, \frac{1}{k(k-1)}\right)$. Provided that $d < \frac{k^2-2k}{1 - k\tau }$, for every $p > \frac{2(k+d)}{d}$ and $\varepsilon > 0$, we have

$$\begin{aligned} \int _{|F_a| \geqslant N^{d/2 - d\tau /2 + \varepsilon } \Vert a\Vert _2 } |F_a|^p \mathrm {d}m\lesssim _{p,\varepsilon } N^{\frac{dp}{2} - (k+d)} \Vert a \Vert _2^p. \end{aligned}$$

Note that lifting this result to a complete restriction estimate via Lemma 3.6 would yield the same result as Corollary 7.2 with a more restrictive condition on *d*, therefore we do not carry out this process. Our method of proof follows again the number-theoretic approach of Bourgain [3] for the parabola, this time in a fashion closer to the original. Remarkably, this approach does not break down when using the weaker minor arc estimates available for the Weyl sums (7.3) associated to the *k*-paraboloid. As in that reference, we first obtain a version of the desired estimate which an extra factor $N^\varepsilon$, whose proof is simpler and serves as a blueprint for the more technical $\varepsilon$-free case. We fix at the outset a sequence $a : \mathbb {Z}^d \rightarrow \mathbb {C}$ supported on $[-N,N]^d$ with $\Vert a \Vert _2 = 1$, and we reuse the notation introduced in Sect. 7. In particular we work again with the exponential sums (7.2) and (7.3), and we fix again a Weyl exponent $\tau = \max \left( 2^{1-k}, \frac{1}{k(k-1)}\right)$.

### Bounds on major and minor arc pieces of the exponential sum

For each dyadic integer *Q* and integer $s \geqslant 0$ such that $1 \leqslant Q \leqslant 2^s$, we define a piece of our original exponential sum by

8.1 $$\begin{aligned} F_{Q,s}(\alpha ,\varvec{\theta }) = F(\alpha ,\varvec{\theta }) \Big [ \Phi _{Q,s}(\alpha ) - \frac{\int \Phi _{Q,s}}{\int \rho } \rho (\alpha ) \Big ]. \end{aligned}$$

By comparison with the simpler definition (7.6), the second term in the parenthesis ensures that $F_{Q,s}$ satisfies good Fourier bounds at non-zero frequencies. However, there is a trade-off in the sense that we only get acceptable physical-side bounds on $F_{Q,s}$ for suffficiently small dimensions, as the next proposition shows.

#### Proposition 8.2

Suppose that $d < \frac{k^2 - 2k}{1 - k\tau }$. We have, uniformly for $Q \leqslant 2^s \leqslant N$,

$$\begin{aligned} \Vert F_{Q,s} \Vert _\infty \lesssim _{\varepsilon } \bigg ( \frac{2^s}{Q} \bigg )^{\frac{d}{k}} Q^{\varepsilon } N^{d(1-\frac{1}{k})}. \end{aligned}$$

#### Proof

From the definitions (7.6) and (8.1), we have

$$\begin{aligned} F_{Q,s}(\alpha ,\varvec{\theta }) = F^{Q,s}(\alpha ,\varvec{\theta }) - \frac{\int \Phi _{Q,s}}{\int \rho } \rho (\alpha ) F(\alpha ,\varvec{\theta }). \end{aligned}$$

By Propositions 7.3 and 7.5, and inserting the bounds (6.8) and (6.10), we obtain

$$\begin{aligned} |F_{Q,s}(\alpha ,\varvec{\theta })| \lesssim \Big ( \frac{2^s}{Q} \Big )^{\frac{d}{k}} Q^{\varepsilon } N^{d - \frac{d}{k}} + \frac{Q}{2^s} \cdot \frac{Q}{N} \cdot N^{d - (k - 2 + d\tau - \varepsilon )}. \end{aligned}$$

Since $Q \leqslant 2^s \leqslant N$ and $(k-2)/(k^{-1}-\tau ) > d$, the second term in the last line may be absorbed into the first for $\varepsilon$ small enough. $\square$

In the rest of this section, we assume that the hypothesis $d < \frac{k^2 - 2k}{1 - k\tau }$ of Theorem 8.1 is satisfied to avoid repetition. We also introduce a technical device analogous to that of Sect. 4 to ensure that all Fourier transforms under consideration stay inside an $N \times \dots \times N \times N^k$ box. We fix a trigonometric polynomial $\psi _N$ on $\mathbb {T}^{d+1}$ such that

$$\begin{aligned}{}[-d(2N)^k,d(2N)^k] \times [-2N,2N]^d \prec \widehat{\psi }_N \prec [-2d(2N)^k,2d(2N)^k] \times [-4N,4N]^d, \end{aligned}$$

which in particular implies that $\int _{\mathbb {T}^{d+1}} \psi _N = 1$. When $H : \mathbb {T}^{d+1} \rightarrow \mathbb {C}$ is a bounded measurable function, we write $\dot{H} = H *\psi _N$ for brevity; note that $\Vert \dot{H} \Vert _p \leqslant \Vert H \Vert _p$ for any $p \geqslant 1$ by Young’s inequality, and that $F = \dot{F}$ by (7.5) and Fourier inversion. With this notation in place, we derive a Fourier estimate improving on that of Proposition 7.6, by exploiting the pseudorandomness of the weight $\Phi _{Q,s} - \frac{\int \Phi _{Q,s}}{\int \rho } \rho$.

#### Proposition 8.3

Uniformly in $(m,\varvec{\ell }) \in \mathbb {Z}^{d+1}$, we have

$$\begin{aligned} |\widehat{\dot{F_{Q,s}}}(m,\varvec{\ell })|&\lesssim _\varepsilon 1_{|m| \lesssim N^k, |\varvec{\ell }| \lesssim N} \Big ( \frac{Q}{2^s N^{k-1}} d( m - |\varvec{\ell }|_k^k,2Q) + \frac{Q^2}{N^{2(k-1)-\varepsilon }} \Big ), \end{aligned}$$

In particular, we have

$$\begin{aligned} \Vert \widehat{\dot{F_{Q,s}}} \Vert _\infty&\lesssim _\varepsilon \frac{Q}{2^s N^{k-1-\varepsilon }}. \end{aligned}$$

#### Proof

Let $\Psi _{Q,s} = \Phi _{Q,s} - \frac{\int \Phi _{Q,s}}{\int \rho } \rho$ and note that $\widehat{\Psi }_{Q,s}(0) = 0$. By a computation similar to that in Proposition 7.6, we find that for any $(m,\varvec{\ell }) \in \mathbb {Z}^{d+1}$,

$$\begin{aligned} \widehat{\dot{F}_{Q,s}}(m,\varvec{\ell }) = \widehat{\psi _N}(m,\varvec{\ell }) \omega _d(\varvec{\ell }) \widehat{\Psi }_{Q,s}( |\varvec{\ell }|_k^k - m ) 1_{ m \ne |\varvec{\ell }|_k^k}. \end{aligned}$$

It then suffices to insert the estimates (6.9) as well as (6.8), (6.10) and (6.11). $\square$

We again define a piece $F_\mathfrak {M}$ of our exponential sum corresponding to the union of all major arcs, and a piece $F_\mathfrak {m}$ corresponding to the minor arcs, this time by

8.2 $$\begin{aligned} F_{\mathfrak {M}} = \sum _{Q \leqslant N_1} \, \sum _{Q \leqslant 2^s \leqslant N} F_{Q,s}, \qquad F_{\mathfrak {m}} = F - F_{\mathfrak {M}}. \end{aligned}$$

#### Proposition 8.4

We have

8.3 $$\begin{aligned} \Vert F_{\mathfrak {m}} \Vert _\infty \lesssim _{\varepsilon } N^{d - d\tau + \varepsilon }. \end{aligned}$$

#### Proof

Recalling the definitions (8.1) and (6.7), we have

$$\begin{aligned} F_{\mathfrak {m}}(\alpha ,\varvec{\theta })&= F(\alpha ,\varvec{\theta }) \bigg [ 1 - \sum _{Q \leqslant N_1} \sum _{Q \leqslant 2^s \leqslant N} \Big ( \Phi _{Q,s}(\alpha ) - \frac{\int \Phi _{Q,s}}{\int \rho } \rho (\alpha ) \Big ) \bigg ] \\&= \rho (\alpha ) F(\alpha ,\varvec{\theta }) \Bigg ( 1 + \sum _{Q \leqslant N_1} \sum _{Q \leqslant 2^s \leqslant N} \frac{\int \Phi _{Q,s}}{\int \rho } \Bigg ). \end{aligned}$$

From (6.8) and (6.10), we deduce that

$$\begin{aligned} |F_{\mathfrak {m}}(\alpha ,\varvec{\theta })|&\lesssim \rho (\alpha ) |F(\alpha ,\varvec{\theta })| \Bigg ( 1 + \sum _{Q \leqslant N_1} \, \sum _{Q \leqslant 2^s \leqslant N} \frac{Q^2}{2^s N^{k-1}} \Bigg ) \\&\lesssim \rho (\alpha ) |F(\alpha ,\varvec{\theta })| \Bigg ( 1 + \frac{1}{N^{k-1}} \sum _{Q \leqslant N'} Q \Bigg ) \\&\lesssim \rho (\alpha ) |F(\alpha ,\varvec{\theta })| \end{aligned}$$

since $\sum _{Q \leqslant N'} Q \lesssim N' \leqslant N^{k-1}$. It remains to insert the bound of Proposition 7.3 to conclude the proof. $\square$

The previous estimates on $F_{Q,s}$ yield bounds for the operator of convolution with this kernel.

#### Proposition 8.5

Uniformly for $Q \leqslant 2^s \leqslant N$, we have

8.4 $$\begin{aligned} \Vert \dot{F}_{Q,s} *f \Vert _\infty&\lesssim _{\varepsilon } \Big ( \frac{2^s}{Q} \Big )^{\frac{d}{k}} Q^{\varepsilon } N^{ d ( 1 - \frac{1}{k} ) } \Vert f \Vert _1, \end{aligned}$$

8.5 $$\begin{aligned} \Vert \dot{F}_{Q,s} *f \Vert _2&\lesssim _\varepsilon \frac{Q}{2^s N^{k-1-\varepsilon }} \Vert f \Vert _2. \end{aligned}$$

#### Proof

By the same argument as in Proposition 7.7, inserting the estimates of Propositions 8.2 and 8.3 instead, the proposition follows. $\square$

Interpolation at the critical exponent almost completely removes the operator constant, as the next proposition shows.

#### Proposition 8.6

Let $p'_0 = \frac{2(k+d)}{d}$. Uniformly for $Q \leqslant 2^s \leqslant N$ and $p \in (1,2]$, we have

8.6 $$\begin{aligned} \Vert \dot{F}_{Q,s} *f \Vert _{p'} \lesssim _\varepsilon \Big [ \Big ( \frac{2^s}{Q} \Big )^\frac{d}{k} N^{d(1-\frac{1}{k})}\Big ]^{1 - \frac{p'_0}{p'}} N^\varepsilon \Vert f \Vert _{p} \end{aligned}$$

In particular, for $p' = p'_0$ we have

8.7 $$\begin{aligned} \Vert \dot{F}_{Q,s} *f \Vert _{p'_0} \lesssim _\varepsilon N^\varepsilon \Vert f \Vert _{p_0} \end{aligned}$$

#### Proof

Fix parameters $p \in (1,2]$ and $\theta \in (0,1]$ such that

8.8 $$\begin{aligned} \frac{1}{p'} = \frac{1-\theta }{\infty } + \frac{\theta }{2}, \qquad \frac{1}{p} = \frac{1-\theta }{1} + \frac{\theta }{2}. \end{aligned}$$

By interpolation between the estimates of Proposition 8.5, we obtain

$$\begin{aligned} \Vert \dot{F}_{Q,s} *f \Vert _{p'}&\lesssim _\varepsilon N^\varepsilon \cdot \Big ( \frac{2^s}{Q} \Big )^{(1-\theta ) \frac{d}{k} } N^{d(1-\frac{1}{k})(1-\theta )} \cdot \Big ( \frac{Q}{2^s} \Big )^\theta \Big ( \frac{1}{N^{k-1}} \Big )^\theta \cdot \Vert f \Vert _p \\&\lesssim N^\varepsilon \cdot \Big ( \frac{2^s}{Q} \Big )^{ \frac{d}{k} - \frac{d}{k}(1+\frac{k}{d})\theta } \cdot N^{d(1-\frac{1}{k}) - \theta (d(1-\frac{1}{k}) + k(1-\frac{1}{k}) ) } \cdot \Vert f \Vert _p \\&\lesssim N^\varepsilon \cdot \Big [ \Big ( \frac{2^s}{Q} \Big )^{\frac{d}{k}} N^{ d(1-\frac{1}{k})} \Big ]^{1 - \frac{k+d}{d} \theta } \cdot \Vert f \Vert _p. \end{aligned}$$

Since $\theta = \frac{2}{p'}$, we see that $1 - \frac{k+d}{d} \theta = 1 - \frac{p'_0}{p'}$, which yields the desired estimate. $\square$

### $\varepsilon$-Full restriction estimates

In this subsection we derive the upper bound in Theorem 8.1 up to a factor $N^\varepsilon$. We fix a weight function $a : \mathbb {Z}^d \rightarrow \mathbb {C}$ supported in $[-N,N]^d$, and we may assume without loss of generality that $\Vert a \Vert _2 = 1$ in proving that variant of Theorem 8.1. We introduce the usual level set $E_\lambda$ and weighted indicator *f* defined by

$$\begin{aligned} E_{\lambda } = \{ |F_a| \geqslant \lambda \},\qquad f = 1_{E_\lambda } \frac{F_a}{|F_a|}. \end{aligned}$$

Recall that the parameter $\lambda$ takes values in $(0,N^{d/2}]$. The usual Tomas–Stein inequality (3.7) (together with our earlier observation $F = \dot{F}$) becomes

8.9 $$\begin{aligned} \lambda ^2 |E_\lambda |^2 \leqslant \langle \dot{F} *f , f \rangle . \end{aligned}$$

#### Proposition 8.7

Let $\varepsilon > 0$ and $p'_0 = \frac{2(k+d)}{d}$. Uniformly for $\lambda \geqslant N^{d/2 - d\tau /2 + \varepsilon }$, we have

$$\begin{aligned} |E_\lambda | \lesssim _\varepsilon N^\varepsilon \lambda ^{-p'_0}. \end{aligned}$$

#### Proof

Starting from (8.9), and using the triangle and Hölder’s inequalities, we obtain

$$\begin{aligned} \lambda ^2 |E_\lambda |^2&\leqslant | \langle \dot{F}_{\mathfrak {M}} *f , f \rangle | + | \langle \dot{F}_{\mathfrak {m}} *f , f \rangle | \\&\leqslant \sum _{Q \leqslant N'} \, \sum _{Q \leqslant 2^s \leqslant N} | \langle \dot{F}_{Q,s} *f , f \rangle | + \Vert \dot{F}_\mathfrak {m}*f \Vert _\infty \Vert f \Vert _1 \\&\leqslant \sum _{Q \leqslant N'} \, \sum _{Q \leqslant 2^s \leqslant N} \Vert \dot{F}_{Q,s} *f \Vert _{p'_0} \Vert f \Vert _{p_0} + \Vert F_{\mathfrak {m}} \Vert _\infty \Vert f \Vert _1^2. \end{aligned}$$

By (7.10) and (8.7), it follows that

$$\begin{aligned} \lambda ^2 |E_\lambda |^2&\lesssim _\varepsilon \sum _{Q \leqslant N'} \, \sum _{Q \leqslant 2^s \leqslant N} N^\varepsilon \Vert f\Vert _{p_0}^2 + N^{d - d\tau + \varepsilon } \Vert f \Vert _1^2 \\&\lesssim _\varepsilon N^\varepsilon |E_\lambda |^{\frac{2}{p_0}} + N^{d - d\tau + \varepsilon } |E_\lambda |^2. \end{aligned}$$

Assuming that $\lambda \geqslant N^{d/2 - d\tau /2 + \varepsilon }$, we infer that

$$\begin{aligned} |E_\lambda |^{\frac{2}{p'_0}} \lesssim N^\varepsilon \lambda ^{-2} \quad \Rightarrow \quad |E_\lambda | \lesssim N^\varepsilon \lambda ^{-p'_0}. \end{aligned}$$

$$\square $$

The previous level set estimate may be integrated into a truncated $\varepsilon$-full restriction estimate.

#### Proposition 8.8

Let $\varepsilon > 0$. For $p \geqslant p'_0 = \frac{2(k+d)}{d}$, we have

$$\begin{aligned} \int _{ |F_a| \geqslant N^{d/2 - d\tau /2 + \varepsilon } } |F_a|^p \mathrm {d}m\lesssim _\varepsilon N^{\frac{dp}{2} - (k+d) + \varepsilon }. \end{aligned}$$

#### Proof

It suffices to invoke Proposition 8.7 in

$$\begin{aligned} \int _{|F_a| \geqslant N^{d/2 - d\tau /2 + \varepsilon }} |F_a|^p \mathrm {d}m&= p \int _{N^{d/2 - d\tau /2 + \varepsilon }}^{N^{d/2}} \lambda ^{p-1} |E_\lambda | \mathrm {d}\lambda \\&\lesssim _\varepsilon N^\varepsilon \int _1^{N^{d/2}} \lambda ^{p - \frac{2(k+d)}{d} -1 } \mathrm {d}\lambda \\&\lesssim _\varepsilon N^{2\varepsilon } \cdot N^{\frac{dp}{2} - (k+d)}. \end{aligned}$$

$$\square $$

### $\varepsilon$-Free restriction estimates

The goal of this section is to derive Theorem 8.1 in full. While we use propositions from the previous subsection, we do not need the final $\varepsilon$-full estimate of Proposition 8.8. We start by stating a distributional version of Lemma 4.8 (which follows immediately from Markov’s inequality).

#### Lemma 8.9

Let $D,Q,X \geqslant 1$ and $B \in \mathbb {N}$. When $Q \leqslant 2X^{1/B}$, we have

$$\begin{aligned} \#\{ |n| \leqslant X \,:\, d(n,Q) \geqslant D \} \lesssim _{\varepsilon ,B} D^{-B} Q^\varepsilon X. \end{aligned}$$

We tacitly assume that the letter *B* denotes an integer from now on. We may now establish a more precise version of the estimate (8.5), using divisor function bounds.

#### Proposition 8.10

Let $B, D \geqslant 1$. Uniformly for $Q \leqslant N^{k/B}$ and $Q \leqslant 2^s \leqslant N$,

8.10 $$\begin{aligned} \Vert \dot{F}_{Q,s} *f \Vert _2 \lesssim _{\varepsilon ,B} \frac{Q^{1+\varepsilon }}{2^s N^{k-1}} \big ( D \Vert f \Vert _2 + D^{-\frac{B}{2}} N^{\frac{k+d}{2}} \Vert f \Vert _1 \big ). \end{aligned}$$

#### Proof

Note that $I :=\Vert \dot{F}_{Q,s} *f \Vert _2 = \Vert \widehat{\dot{F}}_{Q,s} \widehat{f} \Vert _2$. Via the bounds of Proposition 8.3, we obtain

$$\begin{aligned} I&= \Bigg [ \sum _{\begin{array}{c} |m| \lesssim N^k \\ |\varvec{\ell }| \lesssim N \end{array}} |\widehat{\dot{F}}_{Q,s}(m,\varvec{\ell })|^2 |\widehat{f}(m,\varvec{\ell })|^2 \Bigg ]^{1/2} \\&\lesssim \frac{Q}{2^s N^{k-1}} \Bigg [ \sum _{\begin{array}{c} |m| \lesssim N^k \\ |\varvec{\ell }| \lesssim N \end{array}} d( m - |\varvec{\ell }|_k^k,2Q)^2 |\widehat{f}(m,\varvec{\ell })|^2 \Bigg ]^{1/2} + \frac{Q^2}{2^s N^{2(k-1) - \varepsilon }} \Vert \widehat{f} \Vert _2 \end{aligned}$$

Writing $n = m - |\varvec{\ell }|_k^k$, assuming $Q \leqslant N^{k/B}$ and invoking Lemma 8.9, we obtain

$$\begin{aligned} I&\lesssim _{\varepsilon ,B} \frac{Q}{2^s N^{k-1}} \bigg [ D^2 \Vert \widehat{f} \Vert _2^2 + \Vert \widehat{f} \Vert _\infty ^2 N^d \times \#\{ |n| \lesssim N^k \,:\, d(n,2Q) > D \} \bigg ]^{1/2} \\&\quad + \frac{Q^2}{2^s N^{2(k-1)-\varepsilon }} \Vert f \Vert _2 \\&\lesssim \frac{Q}{2^s N^{k-1}} \Big ( D^2 \Vert f \Vert _2^2 + D^{-B} Q^\varepsilon N^{k+d} \Vert f \Vert _1^2 \Big )^{1/2} + \frac{Q}{2^s N^{k-1}} \cdot \frac{Q}{2^s N^{k-1-\varepsilon }} \Vert f \Vert _2. \end{aligned}$$

Since $Q \leqslant 2^s$, the last term may be absorbed into the first. Finally we obtain

$$\begin{aligned} I \lesssim \frac{Q^{1 + \varepsilon }}{2^s N^{k-1}} \big ( D \Vert f \Vert _2 + D^{-\frac{B}{2}} N^{\frac{k+d}{2}} \Vert f \Vert _1 \big ). \end{aligned}$$

$$\square $$

With this more precise $L^1 + L^2 \rightarrow L^2$ estimate in hand, we proceed to interpolate with the $L^1 \rightarrow L^\infty$ estimate as before.

#### Proposition 8.11

Let $B,D \geqslant 1$. Let $p'_0 = \frac{2(k+d)}{d}$ and $p' \in (2,\infty )$. Uniformly for $Q \leqslant N^{k/B}$ and $Q \leqslant 2^s \leqslant N$, we have

$$\begin{aligned} \Vert \dot{F} *f \Vert _{p'} \lesssim _{\varepsilon ,B} Q^\varepsilon \Big [ \Big ( \frac{2^s}{Q} \Big )^{\frac{d}{k}} N^{d(1-\frac{1}{k})} \Big ]^{1 - \frac{p'_0}{p'}} \big ( D^{\frac{2}{p'}} \Vert f \Vert _p + D^{-\frac{B}{p'}} N^{\frac{k+d}{p'}} \Vert f \Vert _1 \big ). \end{aligned}$$

#### Proof

Consider the real number $\theta \in (0,1)$ such that (8.8) holds. By convexity of $L^p$ norms, we have

$$\begin{aligned} I :=\Vert \dot{F}_{Q,s} *f \Vert _{p'} \leqslant \Vert \dot{F}_{Q,s} *f \Vert _{\infty }^{1 - \theta } \Vert \dot{F}_{Q,s} *f \Vert _2^\theta . \end{aligned}$$

Applying (8.4), and (8.10), we obtain

$$\begin{aligned} I&\lesssim _{\varepsilon ,B} Q^\varepsilon \cdot \Big ( \frac{2^s}{Q} \Big )^{(1-\theta )\frac{d}{k}} N^{(1-\theta )d(1-\frac{1}{k})} \cdot \Big ( \frac{Q}{2^s} \Big )^\theta \Big ( \frac{1}{N^{k-1}} \Big )^\theta \\&\times \big ( D^\theta \Vert f \Vert _1^{1-\theta } \Vert f \Vert _2^\theta + D^{-\theta \frac{B}{2}} N^{\theta \frac{k+d}{2}} \cdot \Vert f\Vert _1 ) \end{aligned}$$

Since |*f*| takes values in $\{0,1\}$, we may rewrite this as

$$\begin{aligned} I&\lesssim _{\varepsilon ,B} Q^\varepsilon \Big [ \Big ( \frac{2^s}{Q} \Big )^{\frac{d}{k}} N^{ d(1-\frac{1}{k}) } \Big ]^{1 - \theta \frac{k+d}{d}} \big ( D^\theta \Vert f \Vert _p + D^{ -\theta \frac{B}{2} } N^{ \theta \frac{k+d}{2} } \Vert f \Vert _1 \big ). \end{aligned}$$

The proof is finished upon observing that $\theta = \frac{2}{p'}$ by (8.8), and recalling that $p'_0 = \frac{2(k+d)}{d}$. $\square$

Following the argument of Bourgain [3], we distinguish two cases according to the size of *Q*. We introduce a parameter $Q_1 \geqslant 1$, and we write $F_\mathfrak {M}= F_1 + F_2$ with

8.11 $$\begin{aligned} F_1 = \sum _{Q \leqslant Q_1} \sum _{Q \leqslant 2^s \leqslant N} F_{Q,s}, \qquad F_2 = \sum _{Q_1 < Q \leqslant N_1} \sum _{Q \leqslant 2^s \leqslant N} F_{Q,s}. \end{aligned}$$

#### Proposition 8.12

Suppose that $p' > p'_0$. Let $T \geqslant 1$ and suppose that $1 \leqslant Q_1 \leqslant N^{k/B}$. Then

$$\begin{aligned} \Vert \dot{F}_1 *f \Vert _{p'} \lesssim N^{ d (1 - \frac{p'_0}{p'}) } \big ( T^2 \Vert f \Vert _p + T^{-B} N^{\frac{k+d}{p'}} \Vert f \Vert _1 \big ). \end{aligned}$$

#### Proof

By the triangle inequality and Proposition 8.11 with $T = D^{1/p'}$, it follows that

$$\begin{aligned} \Vert \dot{F}_1 *f \Vert _{p'}&\lesssim \sum _{Q \leqslant Q_1} Q^{\varepsilon - \frac{d}{k} ( 1 - \frac{p'_0}{p'} ) } \sum _{2^s \leqslant N} (2^s)^{ \frac{d}{k} (1-\frac{p'_0}{p'}) } N^{ d ( 1 -\frac{1}{k}) (1 - \frac{p'_0}{p'}) } \\&\cdot \big ( T^2 \Vert f \Vert _p + T^{- B} N^{\frac{k+d}{p'}} \Vert f \Vert _1 \big ). \\&\lesssim N^{ d (1 - \frac{p'_0}{p'}) } \big ( T^2 \Vert f \Vert _p + T^{- B} N^{\frac{k+d}{p'}} \Vert f \Vert _1 \big ). \end{aligned}$$

$$\square $$

We now consider the piece $F_2$ involving large values of the parameter *Q*.

#### Proposition 8.13

Let $p' > p'_0$. We have

$$\begin{aligned} \Vert \dot{F}_2 *f \Vert _{p'} \lesssim N^\varepsilon Q_1^{ - \frac{d}{k} (1 - \frac{p'_0}{p'}) } N^{ d (1-\frac{p'_0}{p'}) } \Vert f \Vert _p. \end{aligned}$$

#### Proof

From the triangle inequality and (8.6), we deduce that

$$\begin{aligned} \Vert \dot{F}_2 *f \Vert _{p'}&\lesssim \sum _{Q > Q_1} Q^{- \frac{d}{k} (1 - \frac{p'_0}{p'}) } \sum _{2^s \leqslant N} (2^s)^{ \frac{d}{k} (1 - \frac{p'_0}{p'}) } \cdot N^\varepsilon N^{ d (1-\frac{1}{k}) (1-\frac{p'_0}{p'} ) } \cdot \Vert f \Vert _p \\&\lesssim N^\varepsilon Q_1^{ - \frac{d}{k} (1 - \frac{p'_0}{p'}) } N^{ d (1-\frac{p'_0}{p'}) } \Vert f \Vert _p. \end{aligned}$$

$$\square $$

#### Proposition 8.14

For $$\frac{2(k+d)}{d} < q \lesssim 1$$,

$$\begin{aligned} |E_\lambda | \lesssim _{\varepsilon ,q} N^{\frac{dq}{2} - (k+d)} \lambda ^{-q} \qquad \text {for }\lambda \geqslant N^{d/2 - d\tau /2 + \varepsilon }. \end{aligned}$$

#### Proof

Starting from (8.9), and recalling the decompositions (8.2) and (8.11), we have, for any $p' > p'_0$,

$$\begin{aligned} \lambda ^2 |E_\lambda |^2&\leqslant |\langle \dot{F}_{\mathfrak {m}} *f , f \rangle | + |\langle \dot{F}_2 *f , f \rangle | + |\langle \dot{F}_1 *f , f \rangle | \\&\leqslant \Vert F_\mathfrak {m}\Vert _\infty \Vert f \Vert _1^2 + \Vert \dot{F}_2 *f \Vert _{p'} \Vert f \Vert _p + \Vert \dot{F}_1 *f \Vert _{p'} \Vert f \Vert _p. \end{aligned}$$

Let $T \geqslant 1$ be a parameter to be determined later, and assume that we have chosen $Q_1$ so that $Q_1 \leqslant N^{k/B}$. Inserting the estimates of Propositions 8.4, 8.12, and 8.13, this yields

$$\begin{aligned} \lambda ^2 |E_\lambda |^2&\lesssim N^{d - d\tau + \varepsilon } |E_\lambda |^2 + N^\varepsilon Q_1^{ - \frac{d}{k} (1-\frac{p'_0}{p'}) } N^{ d (1 - \frac{p'_0}{p'}) } \Vert f \Vert _p^2 \\&+ T^2 N^{ d (1 - \frac{p'_0}{p'}) } \Vert f \Vert _p^2 + T^{-B} N^{ d (1 - \frac{p'_0}{p'}) + \frac{k+d}{p'} } \Vert f \Vert _p \Vert f \Vert _1. \end{aligned}$$

Assume that $\lambda \geqslant N^{d/2 - d\tau /2 + \varepsilon }$ and fix $Q_1 = N^{\varepsilon _1}$, where $\varepsilon _1 = k/2B$. Provided that $\varepsilon$ is small enough, we have then

$$\begin{aligned} \lambda ^2 |E_\lambda |^2 \lesssim T^2 N^{ d - \frac{2(k+d)}{p'} } |E_\lambda |^{2 - \frac{2}{p'}} + T^{-B} N^{ d - \frac{(k+d)}{p'} } |E_\lambda |^{2 - \frac{1}{p'}}. \end{aligned}$$

Writing $\lambda = \eta N^{d/2}$ with $\eta \in (0,1]$, we have either

$$\begin{aligned} |E_\lambda |^{\frac{2}{p'}} \lesssim T^2 N^{ - \frac{2(k+d)}{p'}} \eta ^{-2} \quad \text {or}\quad |E_\lambda |^{\frac{1}{p'}} \lesssim T^{-B} N^{ - \frac{k+d}{p'}} \eta ^{-2}. \end{aligned}$$

Write $D = T^{p'}$, so that in either case

$$\begin{aligned} |E_\lambda | \lesssim D N^{ - (k+d)} \eta ^{-p'} + D^{-B} N^{- (k+d) } \eta ^{-2p'}. \end{aligned}$$

Choose $D = \eta ^{-\nu }$ for parameter $\nu > 0$, so that

$$\begin{aligned} |E_{\lambda }| \lesssim N^{- (k+d) } \eta ^{- p' - \nu } ( 1 + \eta ^{ - p' + (B+1) \nu } ). \end{aligned}$$

Choosing $B \geqslant C/\nu$ with $C > 0$ large enough, we deduce that $|E_\lambda | \lesssim N^{-(k+d)} \eta ^{- p' - \nu }$. Since $q :=p' + \nu$ can be chosen arbitrarily close to $\frac{2(k+d)}{d}$, this concludes the proof, upon recalling that $\eta = \lambda N^{-d/2}$. $\square$

#### Proof of Theorem 8.1

We apply Proposition 8.14 for a certain $\frac{2(k+d)}{d}< q < p$ to obtain

$$\begin{aligned} \int _{|F_a| \geqslant N^{d/2 - d\tau /2 + \varepsilon }} |F_a|^p \mathrm {d}m&= p \int _{N^{d/2 - d\tau /2 + \varepsilon }}^{N^{d/2}} \lambda ^{p-1} |E_\lambda | \mathrm {d}\lambda \\&\lesssim _{p,\varepsilon } N^{\frac{dq}{2} - (k+d)} \int _1^{N^{d/2}} \lambda ^{p - q - 1} \mathrm {d}\lambda . \\&\lesssim _p N^{\frac{dp}{2} - (k+d)}. \end{aligned}$$

$$\square $$

## Appendix A: Bounds on Weyl sums

We fix an integer $k \geqslant 2$. Recall that we defined the Weyl sum *T* by (7.3). In our argument, we make use several times of the following standard minor arc bound.

### Proposition A.1

Let $\tau = \min ( 2^{1-k} , \frac{1}{k(k-1)})$. Suppose that $\alpha \in \mathbb {T}$, $1 \leqslant a \leqslant q$ are such that $|\beta | = \Vert \alpha - \frac{a}{q} \Vert \leqslant \frac{1}{q^2}$ and $N \lesssim q \lesssim N^{k-1}$. For every $\varepsilon > 0$, we have

$$\begin{aligned} |T( \alpha ,\theta )| \lesssim _\varepsilon N^{1 - \tau + \varepsilon } \end{aligned}$$

### Proof

When $\tau = 2^{1-k}$, this is a consequence of Weyl’s inequality [23, Lemma 2.4] (the presence of a smooth weight does not affect the squaring-differencing argument significantly). We let $J_{s,k}(N)$ denote the number of solutions $n_1,\dots ,n_s,m_1,\dots ,m_s \in [N]$ to the system

$$\begin{aligned}&n_1^j + \dots + n_s^j&= m_1^j + \cdots m_s^j&(1 \leqslant j \leqslant k). \end{aligned}$$

The Vinogradov method [23, Theorem 5.2] gives the bound

$$\begin{aligned} |T(\alpha ,\theta )| \lesssim \Big [ (q^{-1} + N^{-k} + q N^{-k}) N^{\frac{1}{2}k(k-1)} J_{s,k-1}(N) \Big ]^{\frac{1}{2s}} \log N, \end{aligned}$$

since the weight $\omega$ is eliminated in the application of the multidimensional sieve [23, Chapter 5]. The latest bound on the Vinogradov mean value [6] gives $J_{s,k-1}(N) \lesssim _\varepsilon N^{2s - \frac{1}{2}k(k-1) + \varepsilon }$ for $s = \frac{1}{2} k(k-1)$. Under our assumptions on *q*, it follows that $|T(\alpha ,\theta )| \lesssim _\varepsilon N^{1-\frac{1}{k(k-1)} + \varepsilon }$. $\square$

On the major arcs, we use a majorant obtained through the Poisson formula and standard bounds on oscillatory integrals and Gaussian sums.

### Proposition A.2

Let $k \geqslant 3$. Suppose that $|\beta | = \Vert \alpha - a/q \Vert \lesssim 1/qN^{k-1}$, $1 \leqslant a \leqslant q \lesssim N$, $(a,q) = 1$. For every $\varepsilon > 0$, we have

$$\begin{aligned} |T(\alpha ,\theta )| \lesssim _\varepsilon q^{-1/k + \varepsilon } \min (N,|\beta |^{-1/k}). \end{aligned}$$

### Proof

Recall that we chose a weight of the form $\omega = \eta ( \frac{\cdot }{N} )$, where $\eta$ is supported on $[-2,2]$. We define a Gaussian sum and an oscillatory integral by

A.1 $$\begin{aligned} S(a,b;q) = \sum _{u \bmod q} e_q( a u^k + b u ), \qquad J( \beta ,\gamma ; N ) = \int _{\mathbb {R}} \eta (x) e( \beta N^k x^k + \gamma N x ) \mathrm {d}x. \end{aligned}$$

Recalling (7.3), writing $\alpha \equiv \frac{a}{q} + \beta \bmod 1$ and summing over residue classes modulo *q*, we obtain

$$\begin{aligned} T(\alpha ,\theta ) = \sum _{u \bmod q} e_q( a u^k ) \sum _{\begin{array}{c} n \in \mathbb {Z}\,: \\ n \equiv u \bmod q \end{array}} \omega (n) e( \beta n^k + \theta n ). \end{aligned}$$

Writing $1_{n \equiv u \bmod q} = q^{-1} \sum _{b \bmod q} e_q(b(u-n))$, we arrive at

$$\begin{aligned} T( \alpha ,\theta ) = \sum _{b \bmod q} q^{-1} S(a,b;q) \sum _{n \in \mathbb {Z}} \omega (n) e( \beta n^k + ( \theta - \tfrac{b}{q} ) n ). \end{aligned}$$

By Poisson’s formula and a change of variable, we deduce that

A.2 $$\begin{aligned} T( \alpha ,\theta ) = \sum _{b \bmod q} q^{-1} S(a,b;q) \sum _{m \in \mathbb {Z}} N J( \beta , \theta - \tfrac{b}{q} - m ; N ). \end{aligned}$$

We write $J( \beta , \theta - \tfrac{b}{q} - m ; N ) = \int _\mathbb {R}\eta (x) e( N \phi _{b,m}(x) ) \mathrm {d}x$, where

$$\begin{aligned} \phi _{b,m}(x) = \beta N^{k-1} x^k + ( \theta - \tfrac{b}{q} - m ) x. \end{aligned}$$

On the support of $\eta$, we have $|x| \leqslant 2$ and therefore

$$\begin{aligned} \phi _{b,m}'(x) = \theta - \tfrac{b}{q} - m + O( \tfrac{1}{q} ) \end{aligned}$$

under our size condition on $\beta$. We fix a large enough constant $C>0$.

For $|m| \geqslant C$, we have $|\phi '_{b,m}| \asymp |m|$ on ${{\mathrm{Supp}}}\eta$, and therefore by stationary phase [21, Chapter VII] we have $| \int _\mathbb {R}\eta e( N \phi _{b,m} ) | \lesssim (N|m|)^{-2}$.

For $\Vert \theta - \frac{b}{q} \Vert \geqslant \frac{C}{q}$, we have $|\phi _{b,m}'| \asymp |\theta - \frac{b}{q} - m| \gtrsim \Vert \theta - \frac{b}{q} \Vert$ on ${{\mathrm{Supp}}}\eta$ and $\Vert \frac{\phi _{b,m}}{|\theta - \frac{b}{q} - m|} \Vert _{C^2} \lesssim 1$, so that by stationary phase again we deduce that $| \int _\mathbb {R}\eta e( N \phi _{b,m} ) | \lesssim (N \Vert \theta - \frac{b}{q} \Vert )^{-1}$.

Finally, for $|m| \leqslant C$ and $\Vert \theta - \frac{b}{q} \Vert \leqslant \frac{C}{q}$, we observe that $|N\phi _{b,m}^{(k)}| \asymp _k |\beta | N^k$ on $\mathbb {R}$, so that by a basic van der Corput estimate [21, Chapter VII], we obtain $| \int _\mathbb {R}\eta e( N \phi _{b,m} ) | \lesssim (1 + |\beta |N^k)^{-1/k}$. For the Gaussian sum, we use a classical bound of Hua [23, Theorem 7.1]: $|q^{-1} S(a,b;q)| \lesssim _\varepsilon q^{-\frac{1}{k} + \varepsilon }$ for $(a,q) = 1$. Inserting these various estimates into (A.2) yields

$$\begin{aligned} |T(\alpha ,\theta )|&\lesssim _\varepsilon q^{-1/k + \varepsilon } \sum _{\begin{array}{c} \Vert \theta - \frac{b}{q} \Vert \leqslant \frac{C}{q} \\ |m| \leqslant C \end{array}} N (1 + |\beta | N^k)^{-\frac{1}{k}} \\&+ q^{-1/k + \varepsilon } \sum _{\begin{array}{c} \Vert \theta - \frac{b}{q} \Vert \geqslant \frac{C}{q} \\ |m| \leqslant C \end{array}} \Vert \theta - \tfrac{b}{q} \Vert ^{-1} \ +\ q^{1 - 1/k +\varepsilon } \sum _{|m| \geqslant C} N^{-1} |m|^{-2} \\&\lesssim q^{-1/k + \varepsilon } N (1 + |\beta | N^k)^{-\frac{1}{k}} \,+\, q^{1 - 1/k + \varepsilon }. \end{aligned}$$

The second term may be absorbed into the first since $|\beta | \lesssim \frac{1}{qN^{k-1}}$ and $1 \leqslant q \lesssim N$, and this concludes the proof. $\square$

## Footnotes

1 That is, when $\mathbf {P}(x_1 + t,\dots ,x_d + t) = \mathbf {P}(x_1,\dots ,x_d)$ for all $x_1,\dots ,x_d,t \in \mathbb {R}$.

2 Formally, $V_{p,Q}$ also depends on $\varepsilon$.
